# Sensing Peroxynitrite in Different Organelles of Murine RAW264.7 Macrophages With Coumarin-Based Fluorescent Probes

**DOI:** 10.3389/fchem.2020.00039

**Published:** 2020-02-20

**Authors:** Maria Weber, Namiko Yamada, Xue Tian, Steven D. Bull, Masafumi Minoshima, Kazuya Kikuchi, Amanda B. Mackenzie, Tony D. James

**Affiliations:** ^1^Department of Chemistry, University of Bath, Bath, United Kingdom; ^2^Centre for Doctoral Training, Centre for Sustainable and Circular Technologies, University of Bath, Bath, United Kingdom; ^3^Department of Material and Life Science, Graduate School of Engineering, Osaka University, Osaka, Japan; ^4^WPI Immunology Frontier Research Center, Osaka University, Osaka, Japan; ^5^Department of Pharmacy and Pharmacology, University of Bath, Bath, United Kingdom; ^6^Centre for Therapeutic Innovation, University of Bath, Bath, United Kingdom

**Keywords:** peroxynitrite, fluorescence, molecular probe, reactive oxygen species, inflammation

## Abstract

The elucidation of biological processes involving reactive oxygen species (ROS) facilitates a better understanding of the underlying progression of non-communicable diseases. Fluorescent probes are a powerful tool to study various ROS and have the potential to become essential diagnostic tools. We have developed a series of coumarin fluorescent probes for the selective and sensitive detection of peroxynitrite (ONOO^−^), a key ROS. Coumarin based probes exhibit good photostability, large Stokes shift and high quantum yields. The three ratiometric probes all contain a boronate ester motif for the detection of ONOO^−^ and a distinctive organelle targeting group. The study of ONOO^−^ generation in a particular organelle will allow more precise disease profiling. Hence, targeting groups for the mitochondria, lysosome and endoplasmic reticulum were introduced into a coumarin scaffold. The three ratiometric probes displayed sensitive and selective detection of ONOO^−^ over other ROS species. All three coumarin probes were evaluated in murine RAW264.7 macrophages for detection of basal and stimulated ONOO^−^ formation.

## Introduction

Macrophages are a diverse population of innate immune cells with roles that include anti-microbial phagocytic activities, wound healing, adipose tissue metabolism and removal of senescent cells (Hirayama et al., 2017). In pathology, macrophages are also linked to the progression of tumors as well as chronic inflammatory diseases and auto-immune conditions such as rheumatoid arthritis (Yang et al., 2018; Di Benedetto et al., 2019). Many macrophage effector functions are regulated or mediated by the formation of reactive oxygen and nitrogen species including phagocytosis, inflammatory responses, pyroptosis and tumor-associated cytotoxicity (Prolo et al., 2014; Fauskanger et al., 2018; Kelley et al., 2019; Wang et al., 2019). In particular, the powerful oxidant peroxynitrite (ONOO^−^) has been shown to play a key role in the control of infections and the regulation of phagocytic activity in macrophages (Prolo et al., 2014). In cellular systems, ONOO^−^ is generated from nitric oxide free radicals (NO·) reacting with superoxide anions (${\text{O}}_{2}^{\cdot -}$) generated by a number of enzymatic pathways including NADPH oxidases. ONOO^−^ can then modify an array of cellular pathways including oxidation and nitration of proteins and lipids. New approaches for site-specific detection of sub-cellular ONOO^−^ will improve our understanding of localized signal transduction and the importance of ONOO^−^ regulation in health and disease.

**Scheme 1:**
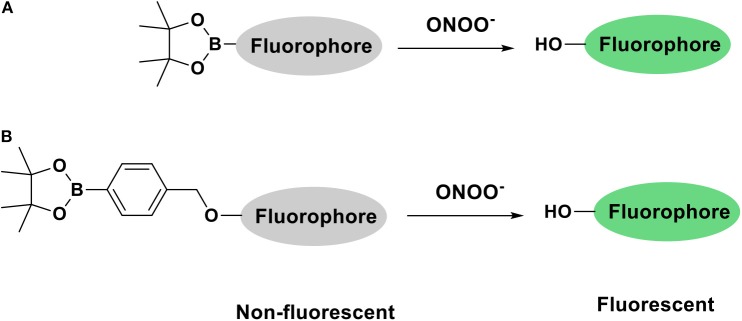
Fluorophore activation by ONOO^−^ triggered cleavage of **(A)** Bpin **(B)** benzyl Bpin.

The development of fluorescent tools to monitor reactive oxygen species (ROS), in particular ONOO^−^, has lead in recent years to the development of a variety of diagnostic small molecule fluorescent probes (Wu et al., 2017; Wang et al., 2018). Our group has exploited boronic acids and esters as a sensing motif for ONOO^−^ (Odyniec et al., 2018; Weber et al., 2018; Wu et al., 2018). The fluorophore is masked by boronate esters which upon reaction with ONOO^−^ results in fluorophore activation and the emission of a distinctive fluorescence signal (Scheme 1). We have previously used resorufin, fluorescein and other fluorophores as core scaffolds (Weber et al., 2018; Wu et al., 2019). More recently, we have been interested in the coumarin scaffold since these are widely employed in a variety of applications, notably in the development of anti-cancer, anti-inflammatory and neurodegenerative drugs (Jameel et al., 2016) as well as fluorescent tools for the detection of biologically relevant analytes such as ROS (Lo and Chu, 2003; Soh et al., 2008; Yuan et al., 2011). Our motivation to use coumarin as a scaffold was its excellent photostability, large Stokes shift and high quantum yield, making them particularly attractive for imaging applications.

A majority of cellular processes linked to ONOO^−^ production and other ROS takes place inside cellular organelles (Zhu et al., 2016). Different strategies of delivering fluorescent probes to specific organelles have been reviewed by Jiang, Chang, Yuan, and co-workers in 2016 (Xu et al., 2016). Herein, we report the synthesis of three fluorescent probes for ONOO^−^ based on a coumarin scaffold. Each probe was designed to detect ONOO^−^ in a different organelle: mitochondrion, lysosome and endoplasmic reticulum *via* the incorporation of relevant targeting groups for these organelles and a boronate ester sensing motif into a coumarin scaffold.

## Results and Discussion

### The Synthesis of Coumarin Based Potential Mitochondria-Targeting (CM), Lysosome-Targeting (CL) and Endoplasmic Reticulum-Targeting (CE) Probes

**Scheme 2:**
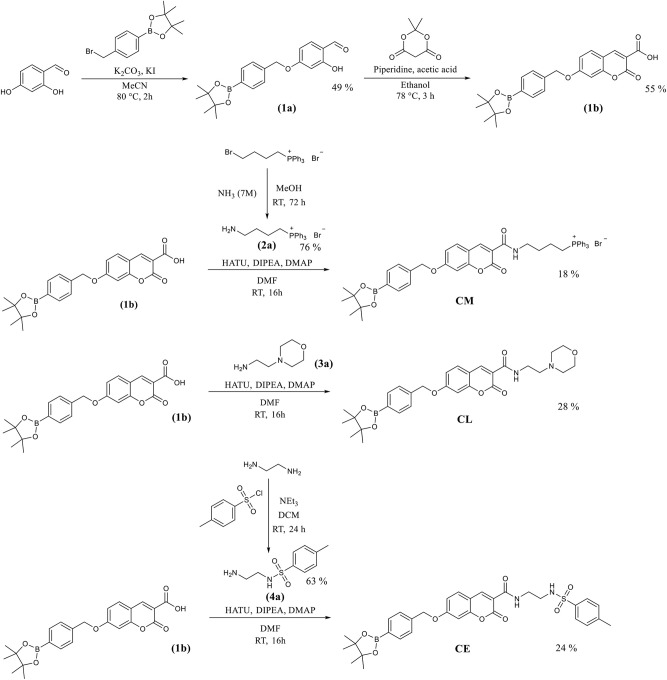
The synthetic routes for the coumarin probes designed to target mitochondria (**CM**), lysosomes (**CL**) or endoplasmic reticulum (**CE**).

The coumarin probes **CM**, **CL**, and **CE** were easily accessible as outlined in Scheme 2. Briefly, 2,4-dihydrobenzaldehyde's alcohol at the 4 position was selectively substituted using 4-(bromomethyl) benzeneboronic acid pinacol ester. This gave **(1a)** containing the benzyl Bpin targeting group in 49% yield. This was followed by the addition of Meldrum's acid to achieve the core structure of coumarin bearing a free carboxylic acid group. **(1b)** Was obtained in 55% yield and required no further purification. **(1b)** Is a key intermediate in the synthesis of our three potential organelle targeting ONOO^−^ molecular probes. To access **CM**, **(1b)** was coupled with **(2a)** which was prepared from (4-bromobutyl) triphenylphosphonium bromide. Similarly, **CE** and **CL** were prepared from **(1b)** by coupling with **(3a)** and **(4a)**. All compounds and intermediates were fully characterized by ^1^H NMR, ^13^C NMR, HRMS, and IR spectroscopy (see Supplementary Material).

### Fluorescence Analysis of the Synthesized Probes

**Figure 1:**
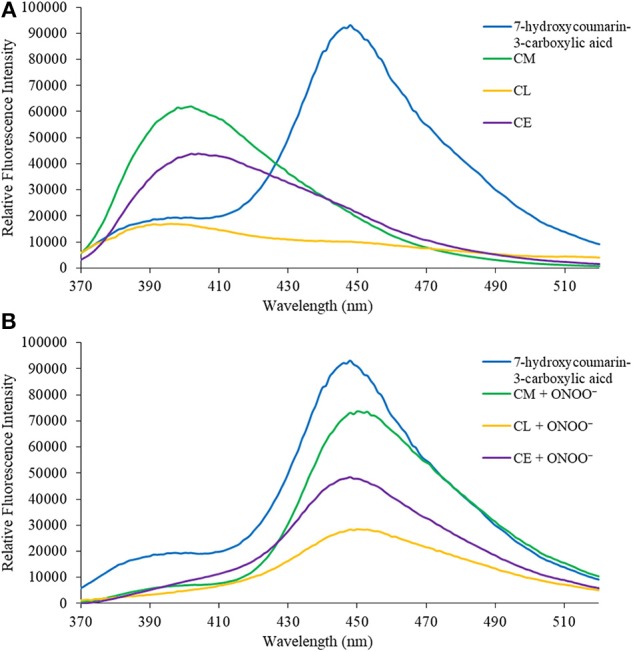
**(A)** Emission spectra for 7-hydroxcoumarin-3-carboxylic acid (5 μM), **CM** (5 μM), **CL** (5 μM), and **CE** (5 μM) in PBS buffer, pH = 7.4 at 25°C. Fluorescence intensities were measured with λ_ex_ = 340 (bandwith: 20) nm on a BMG Labtech CLARIOstar® plate reader. **(B)** Emission spectra for 7-hydroxcoumarin-3-carboxylic acid (5 μM), and **CM** (5 μM), **CL** (5 μM), and **CE** (5 μM) in the presence of ONOO^−^ (50 μM) in PBS buffer, pH = 7.4 at 25°C. Fluorescence intensities were measured with λ_ex_ = 340 (bandwith: 20) nm on a BMG Labtech CLARIOstar® plate reader.

In the first instance, we confirmed that upon reaction with ONOO^−^, **CM**, **CL**, and **CE** generate 7-hydroxycoumarin-3-carboxylic acid. Indicating, that the benzyl Bpin sensing unit was successfully cleaved by the ONOO^−^ (Scheme 1B). Figure 1A illustrates the emission spectrum of **CM**, **CL**, and **CE** without ONOO^−^. All three exhibit moderate fluorescence with maximum emission values at λ_max_ = 400 nm for **CM**, λ_max_ = 395 nm for **CL**, and λ_max_ = 405 nm for **CE**. As expected, 7-hydroxycoumarin-3-carboxylic acid exhibits a maximum emission peak at 447 nm. However, upon addition of ONOO^−^, the emission spectrum of **CM**, **CL** and **CE** changes, and becomes similar to 7-hydroxycoumarin-3-carboxylic acid (Figure 1B), thus indicating the release of 7-hydroxycoumarin-3-carboxylic acid as the fluorescent species of all three organelle targeting probes. Additionally, the probes have the potential to be used as ratiometric probes due to a significant shift of the emission profile of the probes with and without ONOO^−^.

**Figure 2:**
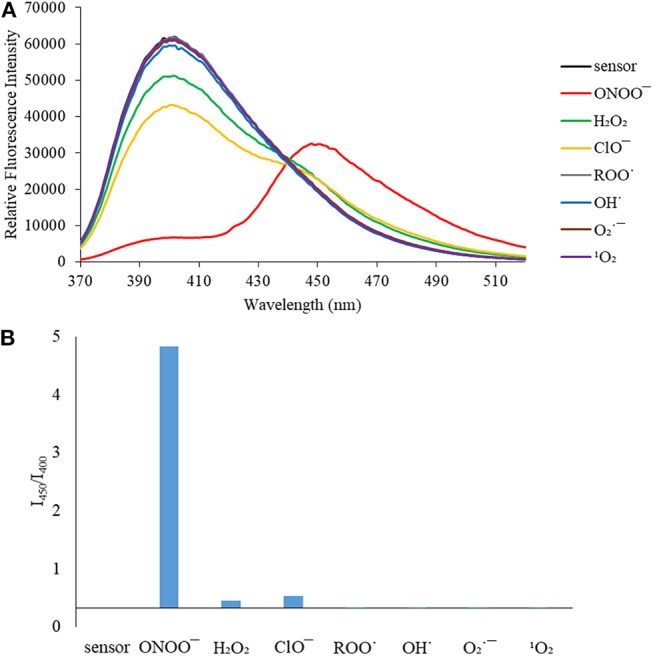
**(A)** Selectivity data for **CM** (5 μM): emission spectrum in the presence of ONOO^−^ (50 μM), OH· (500 μM), ${\text{O}}_{2}^{\cdot -}$ (500 μM), ^1^O_2_ (500 μM) after 5 min. H_2_O_2_ (1 mM), ROO· (500 μM) and ClO^−^ (500 μM) were measured after 30 min. The data was obtained in PBS buffer, pH = 7.4 at 25°C at λ_ex_ = 340 (bandwith: 20) nm on a BMG Labtech CLARIOstar® plate reader. **(B)** Selectivity data for **CM** (5 μM): relative intensity ratio in the presence of ONOO^−^ (50 μM), OH· (500 μM), ${\text{O}}_{2}^{\cdot -}$ (500 μM), ^1^O_2_ (500 μM) after 5 min. H_2_O_2_ (1 mM), ROO· (500 μM) and ClO^−^ (500 μM) were measured after 30 min. The data was obtained in PBS buffer, pH = 7.4 at 25°C at λ_ex_ = 340 (bandwith: 20) nm on a BMG Labtech CLARIOstar® plate reader.

**Figure 3:**
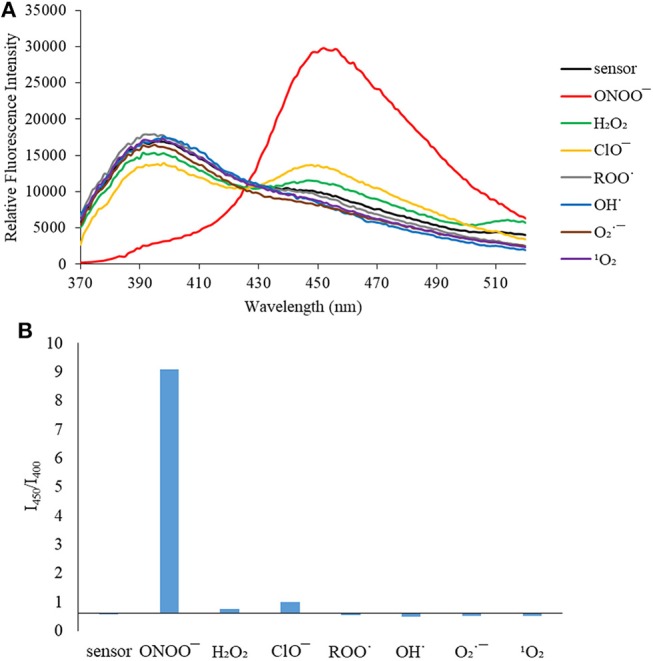
**(A)** Selectivity data for **CL** (5 μM): emission spectrum in the presence of ONOO^−^ (50 μM), OH· (500 μM), ${\text{O}}_{2}^{\cdot -}$ (500 μM), ^1^O_2_ (500 μM) after 5 min. H_2_O_2_ (1 mM), ROO· (500 μM) and ClO^−^ (500 μM) were measured after 30 min. The data was obtained in PBS buffer, pH = 7.4 at 25°C at λ_ex_ = 340 (bandwith: 20) nm on a BMG Labtech CLARIOstar® plate reader. **(B)** Selectivity data for **CL** (5 μM): relative intensity ratio in the presence of ONOO^−^ (50 μM), OH· (500 μM), ${\text{O}}_{2}^{\cdot -}$ (500 μM), ^1^O_2_ (500 μM) after 5 min. H_2_O_2_ (1 mM), ROO· (500 μM) and ClO^−^ (500 μM) were measured after 30 min. The data was obtained in PBS buffer, pH = 7.4 at 25°C at λ_ex_ = 340 (bandwith: 20) nm on a BMG Labtech CLARIOstar® plate reader.

**Figure 4:**
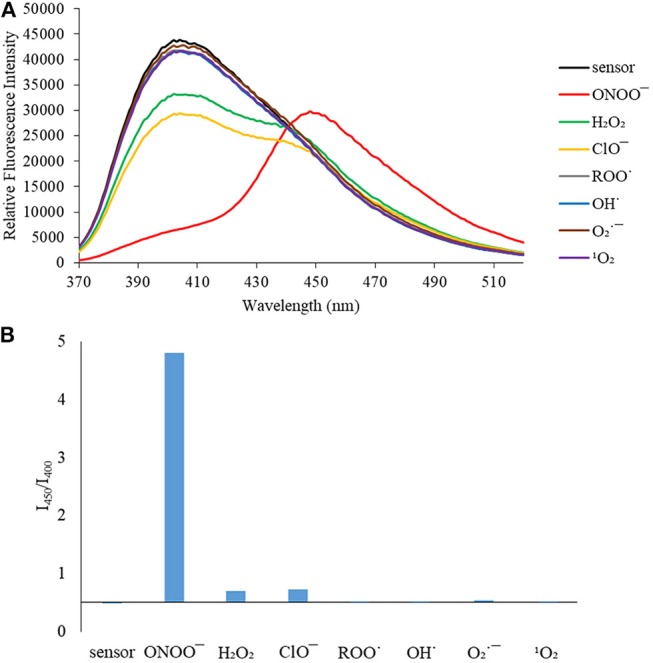
**(A)** Selectivity data for **CE** (5 μM): emission spectrum in the presence of ONOO^−^ (50 μM), OH· (500 μM), ${\text{O}}_{2}^{\cdot -}$ (500 μM), ^1^O_2_ (500 μM) after 5 min. H_2_O_2_ (1 mM), ROO· (500 μM) and ClO^−^ (500 μM) were measured after 30 min. The data was obtained in PBS buffer, pH = 7.4 at 25°C at λ_ex_ = 340 (bandwith: 20) nm on a BMG Labtech CLARIOstar® plate reader. **(B)** Selectivity data for **CE** (5 μM): relative intensity ratio in the presence of ONOO^−^ (50 μM), OH· (500 μM), ${\text{O}}_{2}^{\cdot -}$ (500 μM), ^1^O_2_ (500 μM) after 5 min. H_2_O_2_ (1 mM), ROO (500 μM) and ClO^−^ (500 μM) were measured after 30 min. The data was obtained in PBS buffer, pH = 7.4 at 25°C at λ_ex_ = 340 (bandwith: 20) nm on a BMG Labtech CLARIOstar® plate reader.

**Figure 5:**
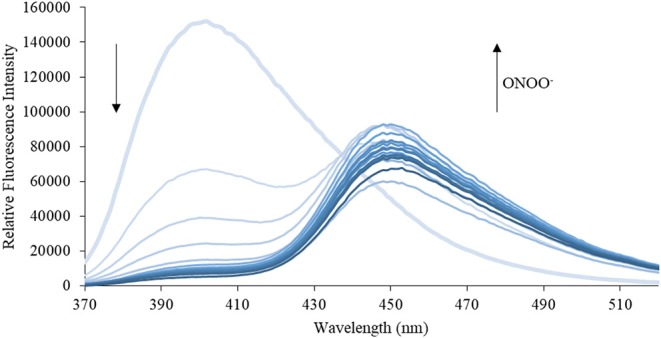
Emission spectra for **CM** (5 μM) in the presence of ONOO^−^ (1, 2, 3, 4, 5, 6, 7, 8, 9, 10, 15, 20, 25, 30, 40, 50 μM) in PBS buffer, pH = 7.4 at 25°C. Fluorescence intensities were measured with λ_ex_ = 340 (bandwith: 20) nm on a BMG Labtech CLARIOstar® plate reader.

**Figure 6:**
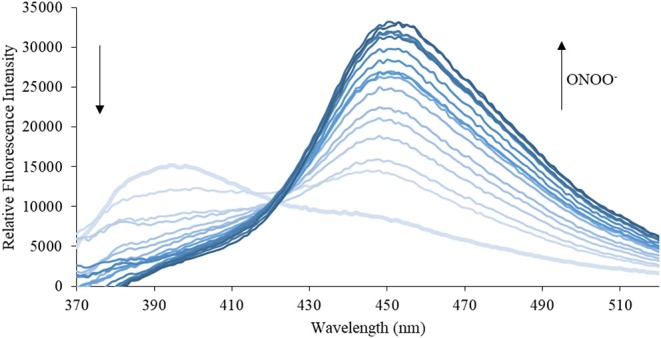
Emission spectra for **CL** (5 μM) in the presence of ONOO^−^ (1, 2, 3, 4, 5, 6, 7, 8, 9, 10, 15, 20, 25, 30, 40, 50 μM) in PBS buffer, pH = 7.4 at 25°C. Fluorescence intensities were measured with λ_ex_ = 340 (bandwith: 20) nm on a BMG Labtech CLARIOstar® plate reader.

**Figure 7:**
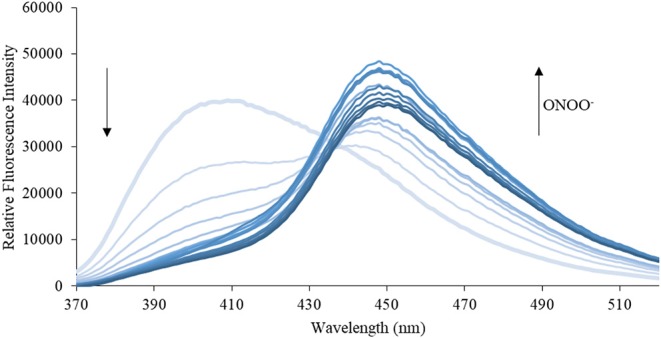
Emission spectra for **CE** (5 μM) in the presence of ONOO^−^ (1, 2, 3, 4, 5, 6, 7, 8, 9, 10, 15, 20, 25, 30, 40, 50 μM) in PBS buffer, pH = 7.4 at 25°C. Fluorescence intensities were measured with λ_ex_ = 340 (bandwith: 20) nm on a BMG Labtech CLARIOstar® plate reader.

We then turned our attention towards ROS selectivity and titrations with ONOO^−^ and H_2_O_2_, plus sensitivity to other ROS. These experiments are to determine the selectivity of **CM**, **CL**, and **CE** for ONOO^−^ and in particular selectivity over H_2_O_2_ since boronate esters are good sensing groups for both species. As illustrated in Figures 2–4, all three probes **CM**, **CL**, and **CE** displayed high selectivity towards ONOO^−^ over H_2_O_2_ and other ROS. All three probes exhibit the same behavior. Surprisingly, H_2_O_2_ has a minimal effect on cleaving the boronic ester moiety demonstrating the strong oxidizing ability of ONOO^−^ for these particular probes. Importantly, the ratiometric nature of probes **CM**, **CL**, and **CE** should allow for the quantitative measurement of ONOO^−^ concentrations. Subsequent titration studies with ONOO^−^ demonstrated that the probes **CM**, **CL**, and **CE** (Figures 5–7) could detect ONOO^−^ (over a range of 1–50 μM) with limits of detection (LoD) determined to be 0.28 μM, 0.26 μM, and 0.36 μM, respectively. Titrations with H_2_O_2_ (see Supplementary Material) confirmed the initial finding of the ROS selectivity study that H_2_O_2_ was not able to release 7-hydroxycoumarin-3-carboxilic acid since no shift in emission occurs. Therefore, with these probes, we were able to show that **CM**, **CL**, and **CE** were particularly selective for ONOO^−^, which is particularly beneficial in a cellular environment to selectively detect ONOO^−^. Therefore, **CM**, **CL**, and **CE** show great promise for the sensitive and selective detection of ONOO^−^ *in situ* over other ROS species. **CM**, **CL**, and **CE** were also shown to be stable over a pH range from 3 to 9 (Figures S21–S23). In addition, all three probes were shown to produce a fluorescent output at relevant physiological pHs: eight for mitochondria, five for lysosome and seven for endoplasmic reticulum (Figures S24–S26).

### Evaluation of CM, CL, and CE for the Detection of ONOO^−^ in RAW 264.7 Macrophages

**Figure 8:**
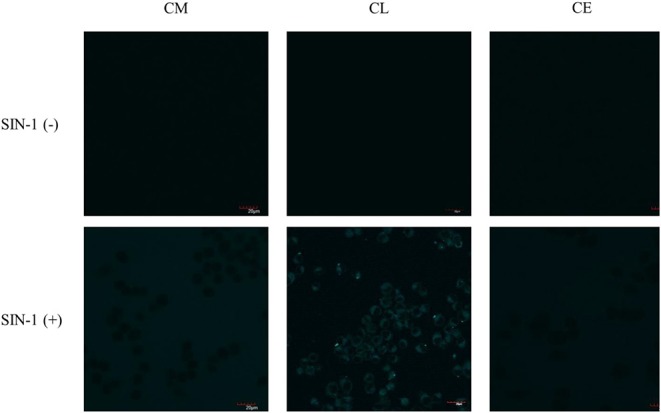
Potential lysosome-targeting **CL** probe detects SIN-1 generated ONOO^−^ in murine macrophages. RAW 264.7 macrophages were stimulated for 1 h with SIN-1 (100 μM). **CM** (20 μM), **CL** (20 μM), and **CE** (20 μM) were then incubated for 30 min, respectively. Blue channel: λ_ex_ = 405 nm, λ_em_ = 420–460 nm. Scale bar: 20 μm. Magnification x60. *n* = 3.

First, exogenous generation of ONOO^−^ was used to validate whether the probes are cell permeable. SIN-1, an ONOO^−^ donor, acts as a positive control to evaluate the probe's ability to detect ONOO^−^ inside the cell. SIN-1 was incubated for 1 h at a concentration of 100 μM, followed by incubation of the relevant probes **CM**, **CL**, and **CE** for 30 min at a concentration of 20 μM (Figure 8). In the absence of SIN-1, no fluorescence signal was detected for all the probes tested (**CM**, **CE**, and **CL**). Upon treatment with SIN-1, no fluorescence signal was detected in RAW264.7 macrophages loaded with **CM** and **CE** probes. We hypothesize that the lack of fluorescence is due to poor cellular uptake of the probes. In contrast, a fluorescence signal was detected in **CL**-loaded, SIN-1 treated macrophages indicating cellular uptake of the **CL** probe and reaction with ONOO^−^.

**Figure 9:**
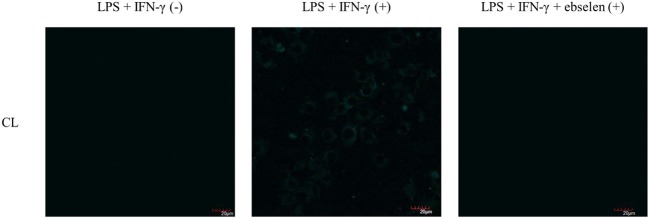
Potential lysosome-targeting **CL** Probe detects ebselen-sensitive LPS and IFN-γ generated ONOO^−^ in murine macrophages. RAW 264.7 macrophages were stimulated with LPS (1 μg/ml) for 4 h and IFN-γ (50 ng/ml) and where indicated treated for 1 h with ebselen (50 μM). **CL** (20 μM), was incubated for 30 min. Blue channel: λ_ex_ = 405 nm, λ_em_ = 420–460 nm. Scale bar: 20 μm. Magnification x60. *n* = 3.

**Figure 10:**
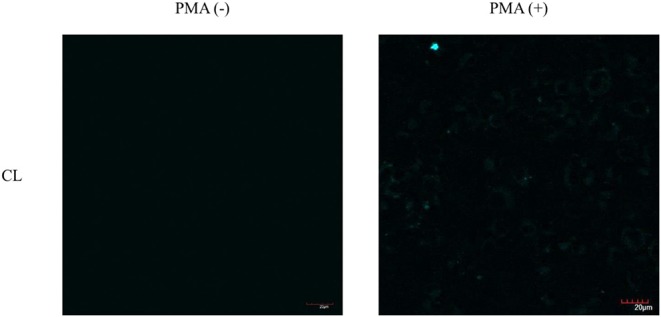
Potential lysosome-targeting **CL** Probe detects PMA generated ONOO^−^ in murine macrophages. RAW 264.7 macrophages were stimulated with PMA (1 μg/ml) for 1 h. **CL** (20 μM), was incubated for 30 min. Blue channel: λ_ex_ = 405 nm, λ_em_ = 420–460 nm. Scale bar: 20 μm. Magnification x60. *n* = 3.

As such, we focused on the characterization of the potential lysosomal targeting **CL** probe and evaluated the ability of **CL** to detect endogenous ONOO^−^ production in RAW264.7 macrophages (Figures 9, 10). To mimic the inflammatory conditions encountered in infections, macrophages were stimulated with lipopolysaccharide (LPS) (1 μg/ml) and interferon (IFN)-γ (50 ng/ml) to trigger the generation of endogenous ONOO^−^. These conditions have been associated with lysosome-localized ONOO^−^ in murine macrophages (Guo et al., 2018). No basal fluorescence was detected in unstimulated macrophages however LPS and IFN-γ elicited an increase in fluorescence in **CL**-loaded macrophages (Figure 9). We predicted that pre-incubation with a ${\text{O}}_{2}^{\cdot -}$ scavenger, ebselen, would attenuate the ONOO^−^-dependent response. It was found that ebselen extinguished the **CL**-fluorescence response induced by LPS and IFN-γ treatment (Figure 9). Finally, we characterized RAW264.7 macrophages with PMA stimulation (Figure 10), which activates ONOO^−^ production in a different way compared to LPS and IFN-γ. PMA activates protein kinase C which stimulates nicotinamide adenine dinucleotide phosphate oxidase (NOX). NOX are a family of enzyme complexes that generate ${\text{O}}_{2}^{\cdot -}$ from molecular oxygen at the expense of NADPH (Rastogi et al., 2016). Hence, PMA can induce enhanced ${\text{O}}_{2}^{\cdot -}$ production via this pathway.

**CL** was able to detect ONOO^−^ via PMA stimulation in RAW 264.7 macrophages. Unfortunately, the weak signal produced by **CL** generated under the various ONOO^−^ cell stimulation conditions, meant that co-localization studies with LysoTracker Green were inconclusive. Indicating that more work will be required to confirm the predicted sub-cellular localization of the **CL** probe.

## Conclusions

A series of organelle targeting ONOO^−^ molecular probes were successfully synthesized, in order to target different organelles (mitochondria, lysosome, and endoplasmic reticulum) and selectively and sensitively detect site specific ONOO^−^ in cellular systems. The ratiometric fluorescent response of all three probes make them interesting candidates for future *in vitro* and *in vivo* studies. Cell studies with RAW 264.7 macrophages revealed possible poor cell permeability of **CM** and **CE**. On the other hand, **CL** was able to detect exogenously and endogenously produced ONOO^−^ in RAW 264.7 macrophages although the fluorescence response of **CL** was relatively weak. We are currently exploring ways to improve the permeability of these probes and enhance the fluorescence response in order to perform co-localization experiments.

## Experimental Section

### Synthesis

#### 2-hydroxy-4-((4-(4,4,5,5-tetramethyl-1,3,2-dioxaborolan-2-yl)benzyl)oxy)benzaldehyde (1a)

2,4-dihydroxybenzaldehyde (1 g, 7.24 mmol) and 4-(bromomethyl)benzeneboronic acid pinacol ester (2.2 g, 7.24 mmol) were dissolved in acetonitrile (15 ml) to which K_2_CO_3_ (1 g, 7.24 mmol) and KI (96 mg, 0.58 mmol) were added. The solution was refluxed for 2 h at 80°C. After completion, the mixture was allowed to cool to RT, diluted with 1 M HCl, extracted with DCM (3 × 10 ml), washed with brine (2 × 20 ml), dried over MgSO_4_, filtered and evaporated *in vacuo*. Recrystallization in ethanol gave 2-hydroxy-4-((4-(4,4,5,5-tetramethyl-1,3,2-dioxaborolan-2-yl)benzyl)oxy)benzaldehyde (1.25 g, 49%) gave a pale orange solid.

^1^H NMR (500 MHz, C*D*Cl_3_): δ = 11.44 (s, 1 H; O–H(8)), 9.71 (s, 1 H; H–C(7)), 7.86 – 7.81 (m, 2 H; Ar*H*), 7.42 (dd, *J* = 9.7, 8.2 Hz, 3 H; Ar*H*), 6.60 (dd, *J* = 8.7, 2.4 Hz, 1 H; Ar*H*), 6.49 (d, *J* = 2.3 Hz, 1 H; Ar*H*), 5.13 (s, 2 H; H–C(7′)), 1.35 ppm (s, 9 H; H–C(2″), H–C(3″),H–C(5″),H–C(6″));^13^C NMR (126 MHz, C*D*Cl_3_): δ = 194.54, 165.97, 164.57, 138.84, 135.44, 135.31, 126.70, 126.55, 115.51, 109.06, 101.89, 84.06, 70.42, 25.02 ppm; m.p.: 80–83°C; IR (ATR): υ = 2978 (br. m, H–C=O), 1649 (s, C=O), 1119 cm^−1^ (s, C–O–C); HR-ESI-MS: m/z (%): 355.1742 ([*M* + H]^+^, calcd for C_20_H_24_O_5_B^+^: 355.1715).

#### 2-oxo-7-((4-(4,4,5,5-tetramethyl-1,3,2-dioxaborolan-2-yl)benzyl)oxy)-2H-chromene-3-carboxylic acid (1b)

2-hydroxy-4-((4-(4,4,5,5-tetramethyl-1,3,2-dioxaborolan-2-yl)benzyl)oxy)benzaldehyde (1 g, 2.82 mmol) and 2,2-dimethyl-1,3-dioxan-4,6-dione (426 mg, 2.96 mmol) were dissolved in ethanol (15 ml) to which piperidine (6 μl, 0.06 mmol) and acetic acid (3.5 μl, 0.06 mmol) were added. The mixture was left to stir first for 30 min at RT and then for 3 h refluxed at 78°C. After completion, the solution was cooled to RT, the precipitate filtered off and dried *in vacuo*. This gave 2-oxo-7-((4-(4,4,5,5-tetramethyl-1,3,2-dioxaborolan-2-yl)benzyl)oxy)-2*H*-chromene-3-carboxylic acid (657 mg, 55%) as a pale yellow solid.

^1^H NMR (500 MHz, DMSO – *d*_6_): δ = 8.65 (s, 1 H; H–C(7)), 7.82 (d, *J* = 8.7 Hz, 1 H; Ar*H*), 7.71 (d, *J* = 7.7 Hz, 2 H; Ar*H*), 7.48 (d, *J* = 7.8 Hz, 2 H; Ar*H*), 7.16 – 7.03 (m, 2 H; Ar*H*), 5.30 (s, 2 H; H–C(7′)), 1.29 ppm (s, 12 H; H–C(2″), H–C(3″), H–C(5″), H–C(6″)); ^13^C NMR (126 MHz, DMSO – *d*_6_): δ = 164.22, 163.17, 157.24, 156.55, 139.37, 134.62, 131.38, 129.02, 127.04, 113.71, 111.93, 105.47, 104.56, 101.18, 83.70, 69.85, 24.66 ppm; m.p.: 162 – 165°C; IR (ATR): υ = 1746 (m, C=O), 1355 cm^−1^ (s, C-O-C); HR-ESI-MS: m/z (%): 445.1447 ([*M* + Na]^+^, calcd for C_23_H_23_O_7_B_1_Na^+^: 445.1433).

#### (4-aminobutyl)triphenylphosphonium bromide (2a)

(4-bromobutyl)triphenylphosphonium bromide (250 mg, 0.63 mmol) was dissolved in an ammonia solution (7N, 25 ml). The solution was stirred at RT for 72 h and evaporated *in vacuo*. This gave (4-aminobutyl)triphenylphosphonium bromide as a white solid (158 mg, 76%).

^1^H NMR (500 MHz, CDCl_3_-*d*): δ = 8.01 – 7.57 (m, 15 H; Ar*H*), 3.66 (s, 2 H; H–C(4)), 3.15 (s, 2 H; H–C(1)), 2.27 (s, 2 H; H–C(3)), 1.95 ppm (s, 2 H; H–C(2)); ^13^C NMR (126 MHz, CDCl_3_-*d*): δ = 135.38, 135.36, 134.13, 134.05, 130.88, 130.78, 118.45, 117.76, 39.33, 34.51, 23.17, 22.76 ppm; m.p.: 67 – 69°C; IR (ATR): υ = 1587 cm^−1^ (m, N–H); HR-ESI-MS: m/z (%): 334.1775 ([*M*]^+^, calcd for C_22_H_25_N_1_${\text{P}}_{1}^{+}$: 334.1719).

#### N-(2-aminoethyl)-4-methylbenzenesulfonamide (4a)

Ethylenediamine (0.85 ml, 10.0 mmol) was dissolved in DCM (10 ml) and cooled to 0°C. NEt_3_ (0.41 ml, 30 mmol) was slowly added, followed by 4-methylbenzenesulfonyl chloride (1.42 g, 7.5 mmol) dissolved previously in DCM (10 ml). The solution was allowed to warm up to RT and stirred for 24 h. The solution was further diluted with DCM (50 ml), washed with 15% NaHCO_3_ and water, dried over MgSO_4_, filtered and evaporated *in vacuo*. Recrystallization in ethanol gave *N*-(2-aminoethyl)-4-methylbenzenesulfonamide (1 g, 63%) as a white solid.

^1^H NMR (500 MHz, CDCl_3_-*d*): δ = 7.71 (d, *J* = 8.3 Hz, 2 H; H–C(2), H–C(4)), 7.31 (d, *J* = 7.9 Hz, 2 H; H–C(1), H–C(5)), 2.98 (s, 2 H; H–C(2′)), 2.85 (s, 2 H; H–C(3′)), 2.40 ppm (s, 3 H; H–C(7)); ^13^C NMR (126 MHz, CDCl_3_-*d*): δ = 143.95, 136.60, 130.00, 129.85, 127.28, 127.25, 43.14, 21.70 ppm; m.p.: 105 – 107°C; IR (ATR): υ = 1596 cm^−1^ (m, N–H); HR-ESI-MS: m/z (%): 215.0934 ([*M* + H]^+^, calcd for C_9_H_15_N_2_O_2_${\text{S}}_{1}^{+}$: 215.0910).

#### (4-(2-oxo-7-((4-(4,4,5,5-tetramethyl-1,3,2-dioxaborolan-2-yl)benzyl)oxy)-2H-chromene-3-carboxamido)butyl)triphenylphosphonium (CM)

(4-aminobutyl)triphenylphosphonium bromide (80 mg, 0.24 mmol), HATU (95 mg, 0.25 mmol) and DMAP (15 mg, 0.12 mmol) were dissolved in DMF (5 ml) to which 2-oxo-7-((4-(4,4,5,5-tetramethyl-1,3,2-dioxaborolan-2-yl)benzyl)oxy)-2*H*-chromene-3-carboxylic acid (100 mg, 0.24 mmol) and DIPEA (60 μl, 0.36 mmol). This solution was left to stir for 16 h at RT. After completion, the mixture was diluted with ethyl acetate (15 ml), washed with water (3 × 20 ml) and brine (3 × 20 ml), dried with MgSO_4_, filtered and evaporated *in vacuo*. FC (90:10 Petroleum ether: ethyl acetate) gave (4-(2-oxo-7-((4-(4,4,5,5-tetramethyl-1,3,2-dioxaborolan-2-yl)benzyl)oxy)-2*H*-chromene-3-carboxamido)butyl)triphenylphosphonium (31 mg, 18%) as a yellow oil.

^1^H NMR (500 MHz, C*D*Cl_3_): δ = 8.48 (s, 1 H; H–C(7)), 8.23 – 8.15 (m, 4 H; Ar*H*), 7.87 – 7.75 (m, 4 H; Ar*H*), 7.75 – 7.62 (m, 4 H; Ar*H*), 7.41 (dd, *J* = 8.2, 3.3 Hz, 4 H; Ar*H*), 6.97 – 6.82 (m, 2 H; Ar*H*), 6.55 – 6.49 (m, 4 H; Ar*H*), 5.16 (d, *J* = 6.0 Hz, 2 H; H–C(1″)), 3.38 – 3.29 (m, 2 H; H–C(3′)), 3.00 – 2.82 (m, 2 H; H–C(6′)), 1.65 (m, 2 H; H–C(4′)), 1.44 – 1.35 (m, 2 H; H–C(5′)), 1.33 ppm (s, 12 H; H–C(2^‴^), H–C(3^‴^), H–C(5^‴^), H–C(6^‴^));^13^C NMR (126 MHz, C*D*Cl_3_): δ = 164.21, 162.61, 157.53, 154.81, 149.02, 148.30, 142.82, 135.32, 135.29, 133.63, 133.60, 133.55, 133.52, 130.87, 130.80, 130.72, 130.70, 130.62, 129.62, 126.72, 126.69, 114.34, 113.94, 111.95, 106.76, 101.91, 101.62, 84.02, 70.65, 48.55, 43.20, 39.27, 24.99 ppm; IR (ATR): υ = 1717 (m, C=O), 1358 cm^−1^ (C-O-C); HR-ESI-MS: m/z (%): 738.3135 ([*M*]^+^, calcd for C_45_H_46_O_6_B_1_N_1_P^+^: 737.3192).

#### N-(2-morpholinoethyl)-2-oxo-7-((4-(4,4,5,5-tetramethyl-1,3,2-dioxaborolan-2-yl)benzyl)oxy)-2H-chromene-3-carboxamide (CL)

2-Morpholinoethylamine (31 mg, 0.24 mmol), HATU (95 mg, 0.25 mmol) and DMAP (15 mg, 0.12 mmol) were dissolved in DMF (5 ml) to which 2-oxo-7-((4-(4,4,5,5-tetramethyl-1,3,2-dioxaborolan-2-yl)benzyl)oxy)-2*H*-chromene-3-carboxylic acid (100 mg, 0.24 mmol) and DIPEA (60 μl, 0.36 mmol). This solution was left to stir for 16 h at RT. After completion, the mixture was diluted with ethyl acetate (15 ml), washed with water (3 × 20 ml) and brine (3 × 20 ml), dried with MgSO_4_, filtered and evaporated *in vacuo*. This gave *N*-(2-morpholinoethyl)-2-oxo-7-((4-(4,4,5,5-tetramethyl-1,3,2-dioxaborolan-2-yl)benzyl)oxy)-2*H*-chromene-3-carboxamide (36 mg, 28%) as a yellow solid.

^1^H NMR (500 MHz, C*D*Cl_3_): δ = 8.16 (s, 1 H; H–C(7)), 7.83 – 7.80 (m, 2 H; Ar*H*), 7.43 – 7.40 (m, 2 H; Ar*H*), 7.08 (d, *J* = 8.6 Hz, 1 H; Ar*H*), 6.47 – 6.42 (m, 2 H; Ar*H*), 5.08 (s, 2 H; H–C(1″)), 3.73 – 3.70 (m, 4 H; H–C(6′), H–C(7′)), 3.68 – 3.64 (m, 2 H; H–C(3′)), 2.66 (t, *J* = 6.7 Hz, 2 H; H–C(4′)), 2.52 (dd, *J* = 5.7, 3.7 Hz, 4 H; H–C(5′), H–C(8′)), 1.34 ppm (s, 12 H; H–C(2^‴^), H–C(3^‴^), H–C(5^‴^), H–C(6^‴^));^13^C NMR (126 MHz, C*D*Cl_3_): δ = 166.26, 164.47, 162.96, 139.70, 135.25, 135.19, 135.14, 132.75, 126.76, 126.69, 112.57, 107.13, 102.56, 83.97, 69.98, 67.10, 59.18, 55.16, 53.99 ppm; m.p.: 125–128°C; IR (ATR): υ = 1612 (s, C=O), 1114 cm^−1^ (s, C-O-C); HR-ESI-MS: m/z (%): 535.2614 ([*M* + H]^+^, calcd for C_29_H_36_O_7_N_2_B^+^: 535.2574).

#### N-(2-((4-methylphenyl)sulfonamido)ethyl)-2-oxo-7-((4-(4,4,5,5-tetramethyl-1,3,2-dioxaborolan-2-yl)benzyl)oxy)-2H-chromene-3-carboxamide (CE)

*N*-(2-aminoethyl)-4-methylbenzenesulfonamide (51 mg, 0.24 mmol), HATU (95 mg, 0.25 mmol) and DMAP (15 mg, 0.12 mmol) were dissolved in DMF (5 ml) to which 2-oxo-7-((4-(4,4,5,5-tetramethyl-1,3,2-dioxaborolan-2-yl)benzyl)oxy)-2*H*-chromene-3-carboxylic acid (100 mg, 0.24 mmol) and DIPEA (60 μl, 0.36 mmol). Ths solution was left to stir for 16 h at RT. After completion, the mixture was diluted with ethyl acetate (15 ml), washed with water (3 × 20 ml) and brine (3 × 20 ml), dried with MgSO_4_, filtered and evaporated *in vacuo*. This gave *N*-(2-((4-methylphenyl)sulfonamido)ethyl)-2-oxo-7-((4-(4,4,5,5-tetramethyl-1,3,2-dioxaborolan-2-yl)benzyl)oxy)-2*H*-chromene-3-carboxamide (35 mg, 24%) as a yellow oil.

^1^H NMR (500 MHz, C*D*Cl_3_): δ = 8.17 (s, 1 H; H–C(7)), 7.82 (dq, *J* = 5.0, 3.1, 2.6 Hz, 2 H; Ar*H*), 7.71 – 7.64 (m, 3 H; Ar*H*), 7.45 – 7.37 (m, 2 H; Ar*H*), 7.31 – 7.17 (m, 2 H; Ar*H*), 6.52 – 6.47 (m, 2 H; Ar*H*), 5.18 – 5.13 (m, 2 H; H–C(1″)), 3.02 (d, *J* = 1.3 Hz, 2 H; H–C(3′)), 2.41 (d, *J* = 2.8 Hz, 2 H; H–C(4′)), 2.39 (s, 3 H; H–C(12′)), 1.33 ppm (s, 12 H; H–C(2^‴^), H–C(3^‴^), H–C(5^‴^), H–C(6^‴^));^13^C NMR (126 MHz, C*D*Cl_3_): δ = 154.65, 149.04, 148.76, 143.50, 136.90, 135.28, 130.88, 130.06, 129.82, 128.46, 127.14, 126.71, 126.68, 114.35, 114.28, 113.95, 106.74, 101.88, 101.60, 83.98, 70.78, 43.08, 39.21, 24.98, 21.61 ppm; IR (ATR): υ = 1720 (m, C=O), 1154 cm^−1^ (s, C-O-C); HR-ESI-MS: m/z (%): 619.2263 ([*M* + H]^+^, calcd for C_32_H_36_O_8_N_2_${\text{B}}_{1}^{+}$: 619.2243).

### Fluorescent Characterization of the Probes

The coumarin-based probes were analyzed using a BMG Labtech CLARIOstar plate reader where compounds were added to a Griener Bio-one 96-well, black-walled microplate with f-bottom chimney wells (ThermoFisher). Fluorescent readings were collected using BMG Labtech MARS software. HPLC or fluorescent grade solvents and de-ionized water were used in the measurements. The measurements of pH were performed using a Hanna Instruments HI 9321 microprocessor pH meter calibrated routinely using Fisher Chemicals standard buffer solutions (pH 4.0—phthalate, pH 7.0—phosphate, and pH 10.0—borate). A Perkin-Elmer Lamba20 Sepctrophotmeter was used for UV-Vis measurements where probes were analyzed using a Starna Silica (quartz) cuvette with 10 mm path lengths with two faces polished where data was collected using the Perkin-Elmer UVWinlab software package. For the characterization of probes, freshly prepared phosphate-buffered saline (PBS) contained 52% methanol in water 10 mM KCl, 2.752 mM KH_2_PO_4_ and 2.757 mM Na_2_HPO_4_ (pH 7.4 with 1M HCl (aq)). Stock solutions of ONOO^−^ were prepared immediately prior to use where 3 M NaOH was cooled to 0°C to which 0.7 M H_2_O_2_, 0.6 M NaNO_2_, and 0.6 M HCl were added simultaneously. The freshly prepared ONOO^−^ solution was analyzed spectrophotometrically where ONOO^−^ concentration was estimated through ε = 1,670 ± 50 cm^−1^ M^−1^ at 302 nm in 0.1 M NaOH (aq.). Commercially available hydrogen peroxide (H_2_O_2_) was used where H_2_O_2_ concentration was measured using spectrophotometrical analysis with ε = 43.6 cm^−1^ M^−1^ at 240 nm. The concentration of ^−^OCl in commercially available Sodium hypochlorite (NaOCl) was evaluated using spectrophotometrical measurements with ε = 250 cm^−1^ M^−1^ at 292 nm. H_2_O_2_ (1 mM) was reacted with NaClO (1 mM) to generate ^1^O_2_. H_2_O_2_ was combined and stirred with aq. NaOCl for 2 min. ROO· was prepared from 2, 2′-azobis (2-amidinopropane) dihydrochloride. AAPH (2,2′-azobis (2-amidinopropane) dihydrochloride, 10 M) was added and stirred in de-ionized water at 37°C for 30 min. ${\text{O}}_{2}^{\cdot -}$ was formed from KO_2_ (1 eq) and 18-crown-6 (2.5 eq) dissolved in DMSO. HO· was generated via a Fenton reaction: ferrous chloride (1 M) was combined with 10 eq of H_2_O_2_ (37.0 wt%). ROS/RNS-sensitivity of each probe were determined by titration at 25°C in PBS buffer (pH 7.4). Each probe was studied at 5 μM +with different concentrations of ROS/RNS as indicated in the Figure.

### *In vitro* Characterization in RAW264.7 Macrophages

#### Cell culture

Murine RAW264.7 macrophages were kindly donated by Prof. Masaru Ishii from the Graduate School of Medicine, Osaka University. The cells were grown in complete medium containing high-glucose Dulbecco's modified Eagle medium (DMEM) plus GlutaMAX-I supplemented (ThermoFisher, 31966021) with 10% fetal bovine serum (FBS, ThermoFisher, 16000044), 100 U/mL penicillin and 100 μg/mL streptomycin (ThermoFisher, 15140122) at in a humidified atmosphere with 5% CO_2_. During cell passaging (every 2 days), media was removed, washed with PBS (2 x 4 ml), trypsin (4 ml, ThermoFisher, 15400054) was added and incubated for 3 min at 37°C, 5% CO_2_. The solution was mixed and 1 ml of cell solution transferred to a new culture dish containing 10 ml of complete medium. Prior to the experiment, cells were plated in a glass bottom dish (35 mm Iwaki, I.C.T., S. L., 3930-035) at a cell density of 5 × 10^5^ cells and cultured in 1 ml DMEM medium (without penicillin or streptomycin) for 18 h in a humidified environment at 37°C in 5% CO_2_. The cells were then treated as described in the following sections. Where indicated, the subsequent treatments were performed in phenol red-free DMEM (ThermoFisher, 21041025) with no added FBS, penicillin or streptomycin (described as “phenol red-free DMEM”).

#### Investigating CM, CE, and CL with SIN-1

The culture media was removed, cells were washed twice with Hanks' Balanced Salt Solution (HBSS) (2 × 2 ml) followed by the addition of 100 μM SIN-1 (Cayman Chemical, 82220) in phenol red-free DMEM and incubated for 30 min or 1 h at 37°C in 5% CO_2_, as indicated. The SIN-1 containing DMEM culture media was removed, the cells were washed twice with HBSS (2 × 2 ml), then the probe (CM, CL, or CE) (20 μM) in phenol red-free DMEM was added and incubated for 30 min at 37°C in 5% CO_2_. The cells were then immediately analyzed using fluorescent confocal microscopy.

#### Investigating CL With LPS, IFN-γ, and Ebselen

*Escherichia coli* LPS (1 μg/ml, Wako, 120-05131) and IFN-γ (50 ng/ml, Peprotech, 315-05) were added and incubated in complete culture medium for 4 h at 37°C in 5% CO_2_. The LPS/IFN-γ containing culture media was removed, cells were washed twice with HBSS (2 × 2 ml), (as indicated, ebselen (50 μM, TCI, E0946) was incubated for 1 h prior to addition of the probe), then the CL probe (20 μM) in phenol red-free DMEM was added and incubated for 30 min at 37°C in 5% CO_2_. The cells were then immediately analyzed using fluorescent confocal microscopy.

#### Investigating CL With Phorbol 12-myristate 13-acetate (PMA)

The complete DMEM culture media was removed, cells were washed twice with HBSS (2 × 2 ml), PMA (1 μg/ml, Cayman Chemical, 10008014) in phenol red-free DMEM was added and incubated for 1 h at 37°C in 5% CO_2_. The PMA-containing phenol red-free DMEM was removed, cells were washed twice with HBSS (2 × 2 ml), CL (20 μM) in phenol red-free DMEM was added and incubated for 30 min at 37°C in 5% CO_2_. The cells were then immediately analyzed using fluorescent confocal microscopy.

#### Fluorescent Confocal Microscopy

Fluorescence microscopy images were captured on an Olympus, FLUOVIEW FV10i confocal microscope using 35 mm glass base dishes. Images were captured at a magnification of x60 with the following parameters: λ_ex_ = 405 nm, λ_em_ = 420–460 nm. Laser 405 nm with an intensity of 50% was used. Cells were imaged at 37°C. Processing and analysis of confocal microscopy images were performed with Image J (https://imagej.nih.gov/ij/).

## Data Availability Statement

All data supporting this study are provided as supplementary information accompanying this paper.

## Author Contributions

MW designed and carried out the synthesis, fluorescence and cell studies. NY assisted in the cell studies. XT helped with the fluorescence analysis. MM and KK provided advice on cell studies and provided facilities. SB, AM, and TJ offered guidance on the project. The manuscript was written by MW with support from TJ and the final version was edited and approved by all the contributing authors.

### Conflict of Interest

The authors declare that the research was conducted in the absence of any commercial or financial relationships that could be construed as a potential conflict of interest.
