# Supplementary material for: Sensing Peroxynitrite in Different Organelles of Murine RAW264.7 Macrophages With Coumarin-Based Fluorescent Probes
Source: Front Chem. 2020 Feb 20;8:39. doi: 10.3389/fchem.2020.00039 (PMC7044669; doi:10.3389/fchem.2020.00039)
Supplement: Supplementary file 1 [file Data_Sheet_1.docx]

Sensing peroxynitrite in different organelles of murine RAW264.7 macrophages with coumarin-based fluorescent probes

Maria Weber^1,2*^, Namiko Yamada^3^, Xue Tian^1^, Steven D. Bull^1^, Masafumi Minoshima^3^, Kazuya Kikuchi^3,4^, Amanda B. Mackenzie^5,6^, Tony D. James^1*^

^1^Department of Chemistry, University of Bath, Bath BA2 7AY, UK

^2^Centre for Doctoral Training, Centre for Sustainable & Circular Technologies, University of Bath, Bath BA2 7AY, UK

^3^Department of Material and Life Science, Graduate School of Engineering, Osaka University, 2-1 Yamadaoka, Suita, Osaka, 565-0871, Japan

^4^WPI Immunology Frontier Research Center, Osaka University, 2-1 Yamadaoka, Suita, Osaka 565-0871, Japan

^5^Department of Pharmacy and Pharmacology, University of Bath, Bath BA2 7AY, UK

^6^Centre for Therapeutic Innovation, University of Bath, Bath BA2 7AY, UK

Supplementary Material

[1 NMR 2](#_Toc22127099)

[2 Fluorescence 16](#_Toc22127100)

# NMR

| 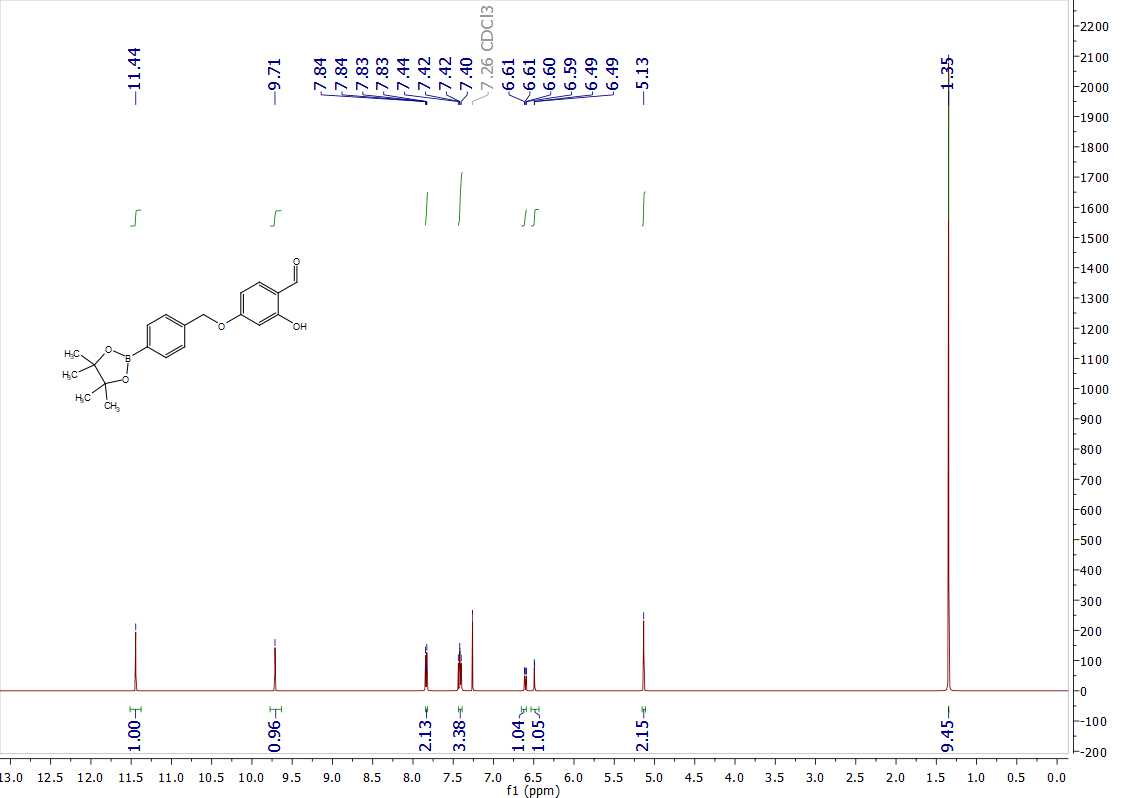 |
| --- |

Figure S 1: 2-hydroxy-4-((4-(4,4,5,5-tetramethyl-1,3,2-dioxaborolan-2-yl)benzyl)oxy)benzaldehyde (1a) – ^1^H NMR

| 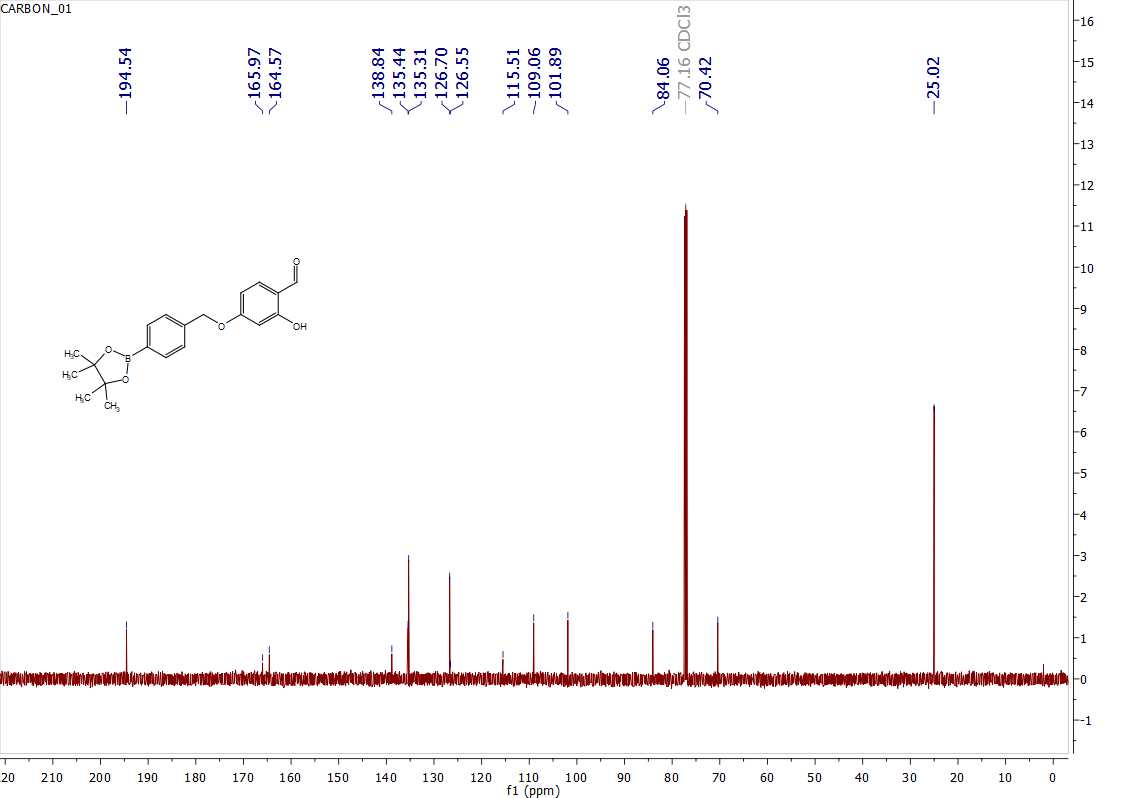 |
| --- |

Figure S 2: 2-hydroxy-4-((4-(4,4,5,5-tetramethyl-1,3,2-dioxaborolan-2-yl)benzyl)oxy)benzaldehyde (1a) – ^13^C NMR

| 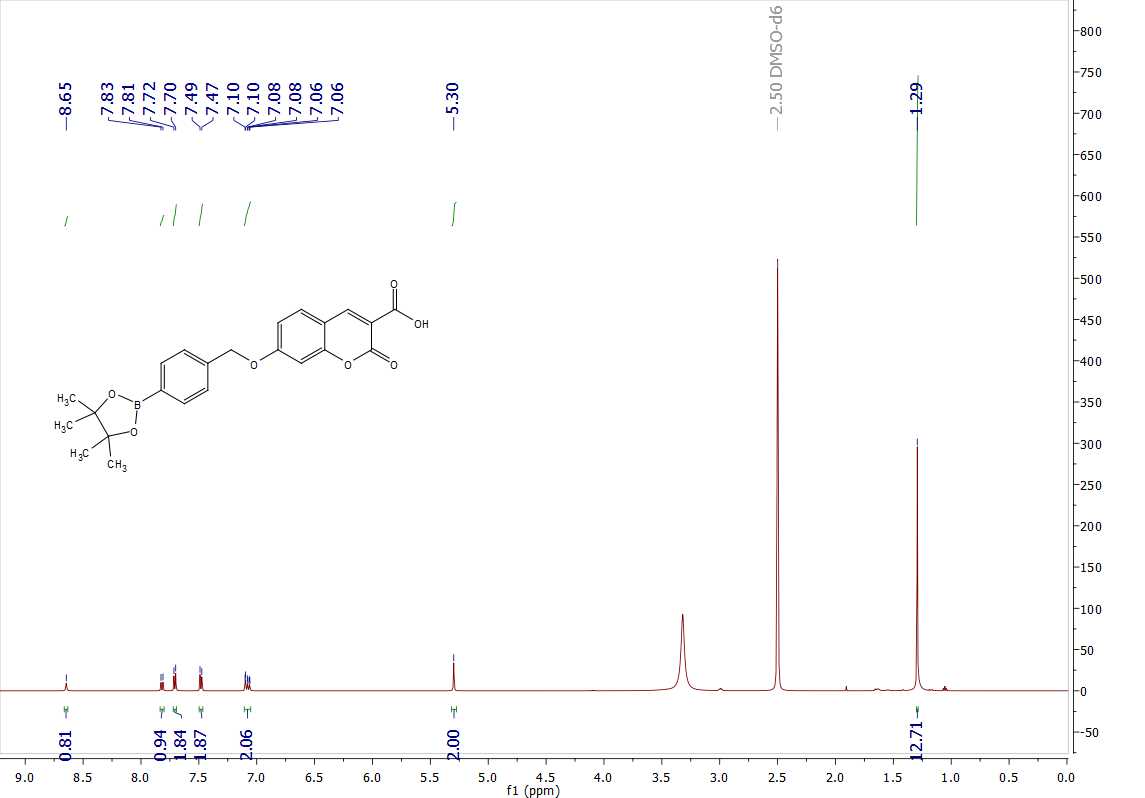 |
| --- |

**Figure S 3:** 2-oxo-7-((4-(4,4,5,5-tetramethyl-1,3,2-dioxaborolan-2-yl)benzyl)oxy)-2*H*-chromene-3-carboxylic acid (1b) – ^1^H NMR

| 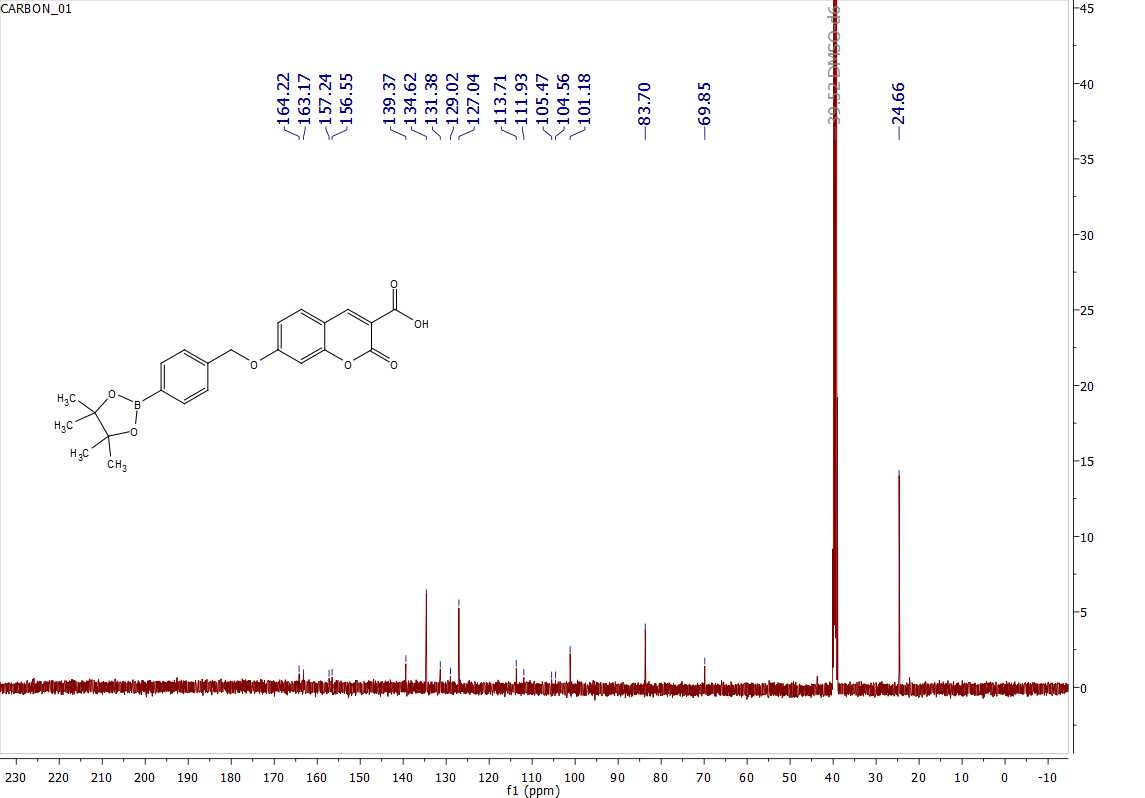 |
| --- |

Figure S 4: 2-oxo-7-((4-(4,4,5,5-tetramethyl-1,3,2-dioxaborolan-2-yl)benzyl)oxy)-2*H*-chromene-3-carboxylic acid (1b) – ^13^C NMR

| 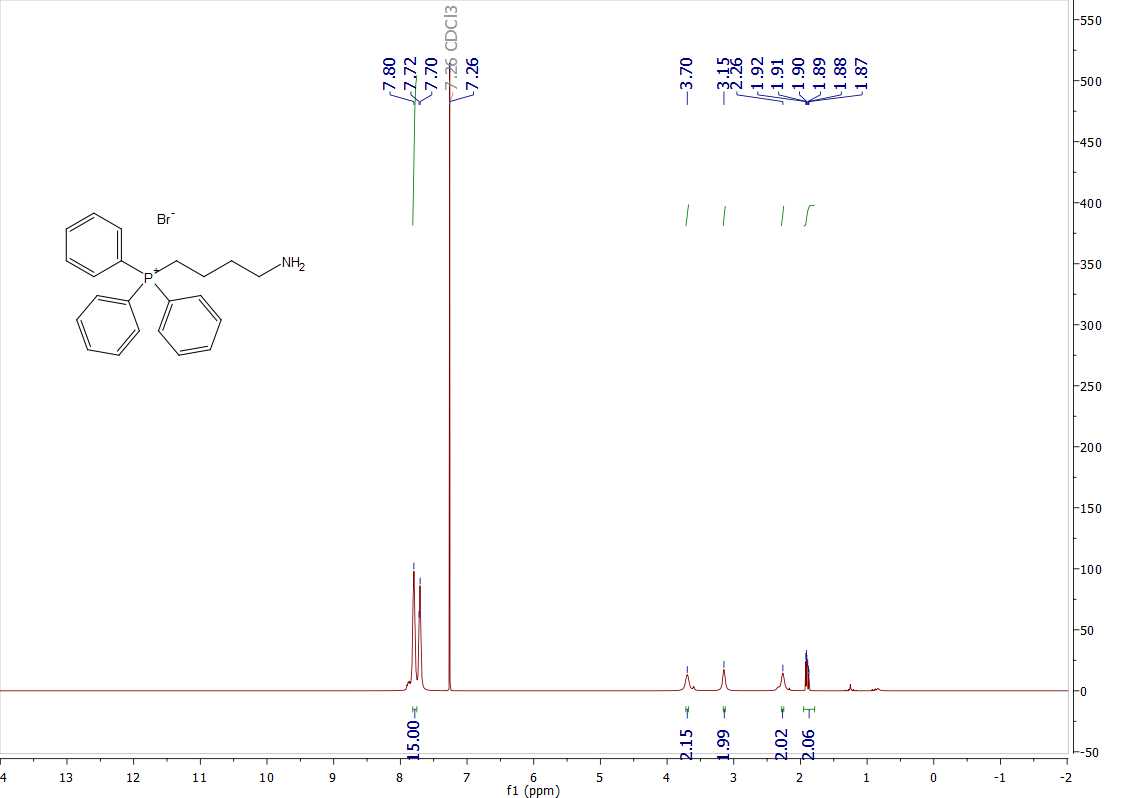 |
| --- |

Figure S 5: (4-aminobutyl)triphenylphosphonium bromide (2a) – ^1^H NMR

| 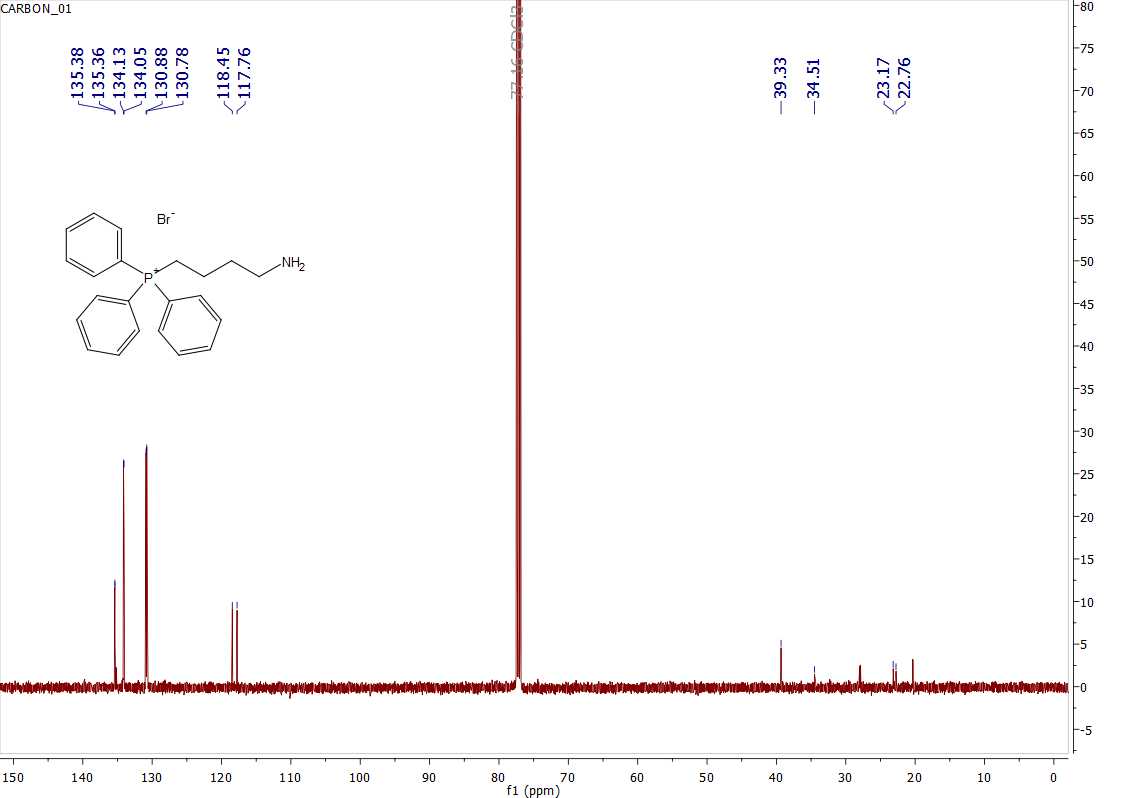 |
| --- |

Figure S 6: (4-aminobutyl)triphenylphosphonium bromide (2a) – ^13^C NMR

| 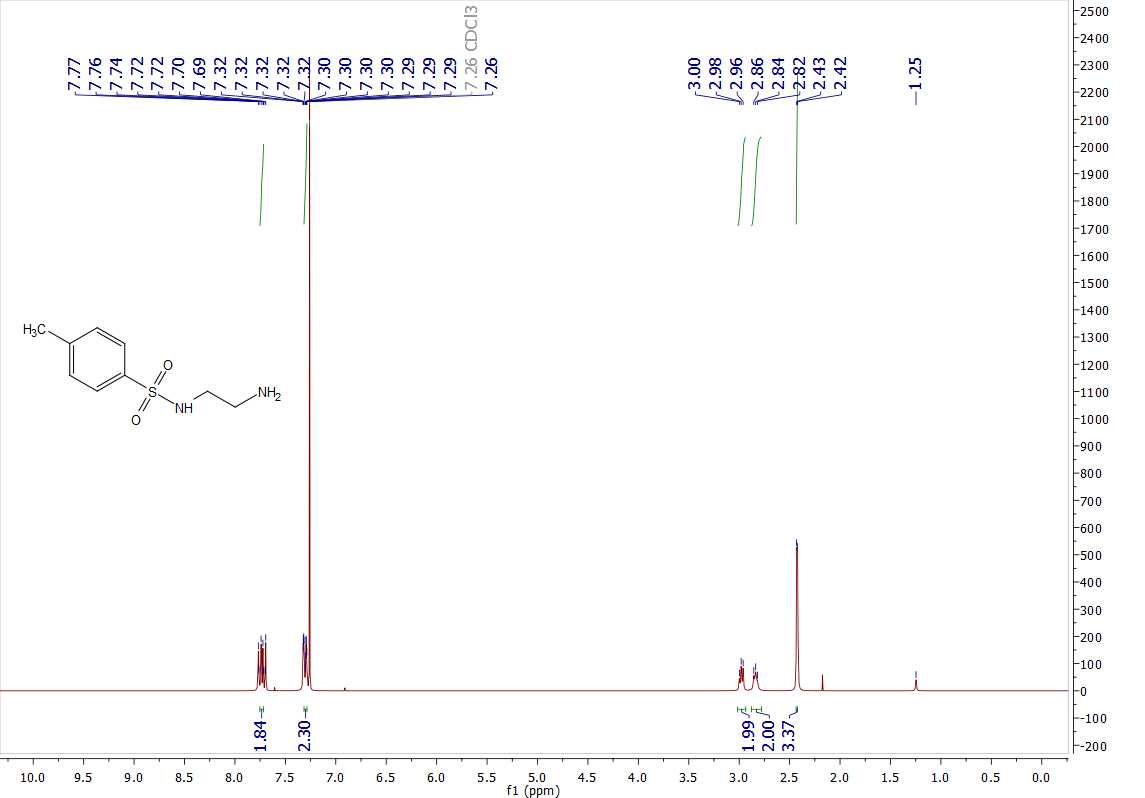 |
| --- |

Figure S 7: *N*-(2-aminoethyl)-4-methylbenzenesulfonamide (4a) – ^1^H NMR

| 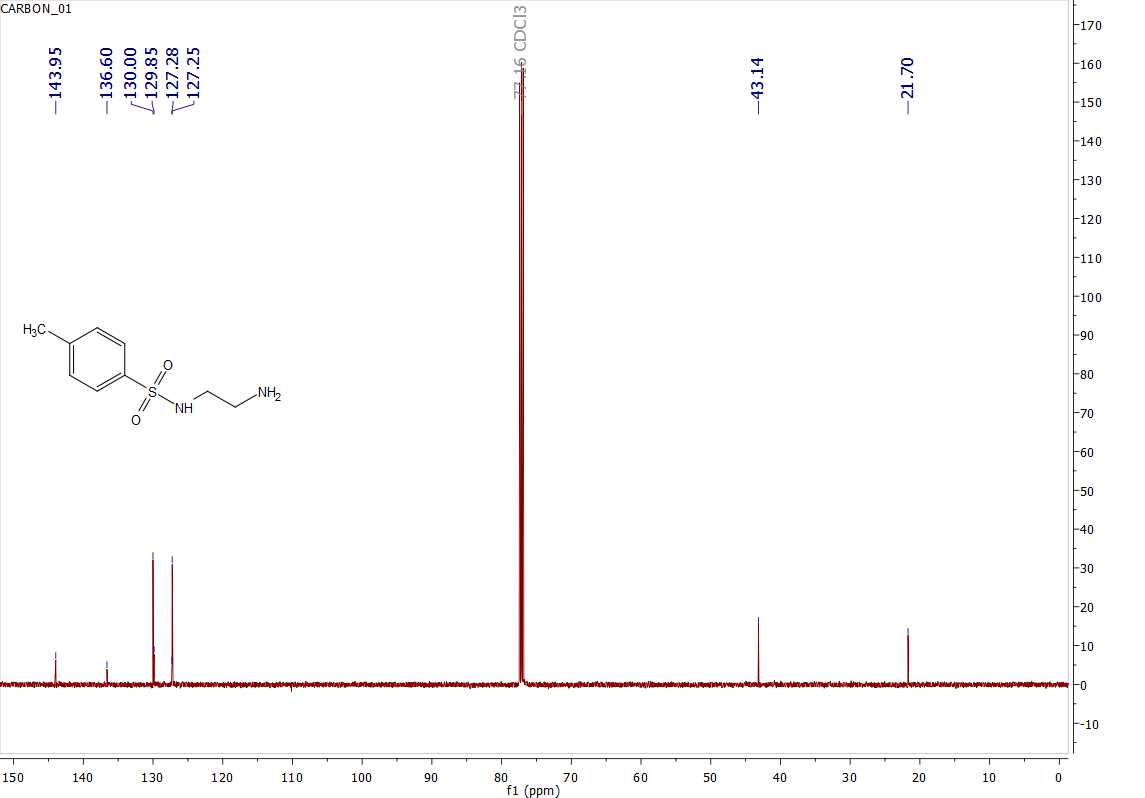 |
| --- |

Figure S 8: *N*-(2-aminoethyl)-4-methylbenzenesulfonamide (4a) – ^13^C NMR

| 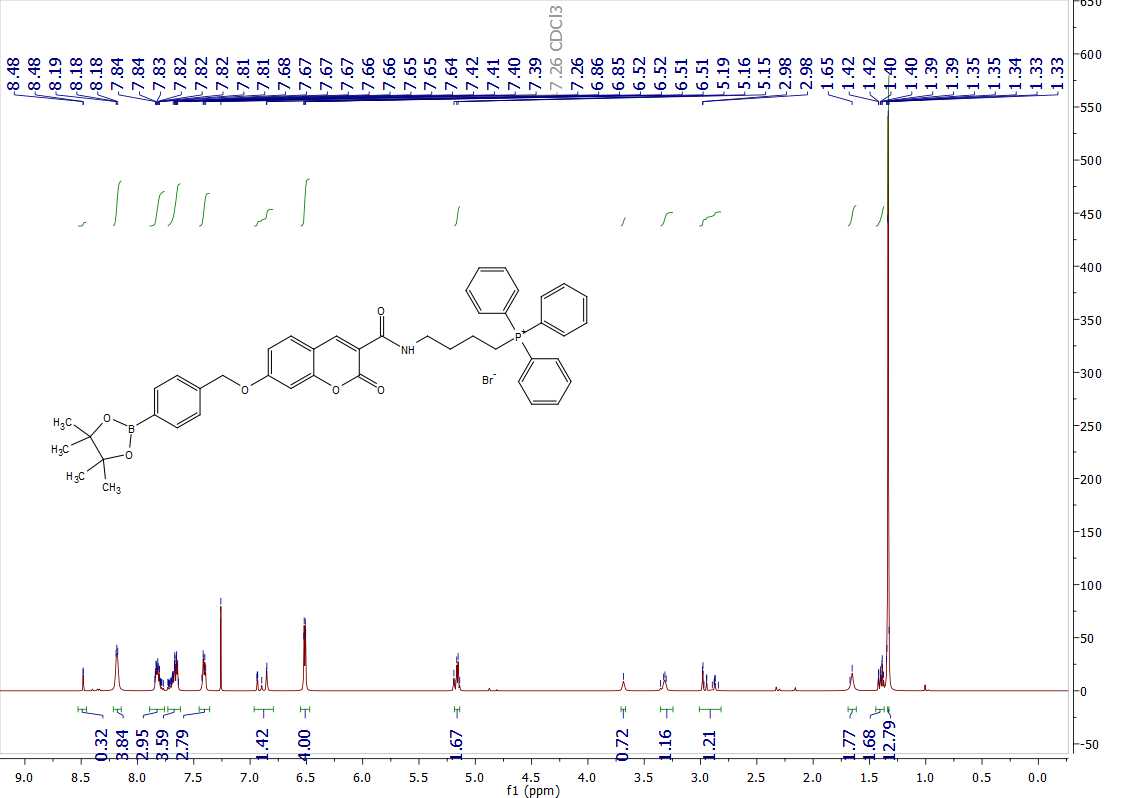 Figure S 9: (4-(2-oxo-7-((4-(4,4,5,5-tetramethyl-1,3,2-dioxaborolan-2-yl)benzyl)oxy)-2*H*-chromene-3-carboxamido)butyl)triphenylphosphonium (CM) – ^1^H NMR |
| --- |
| 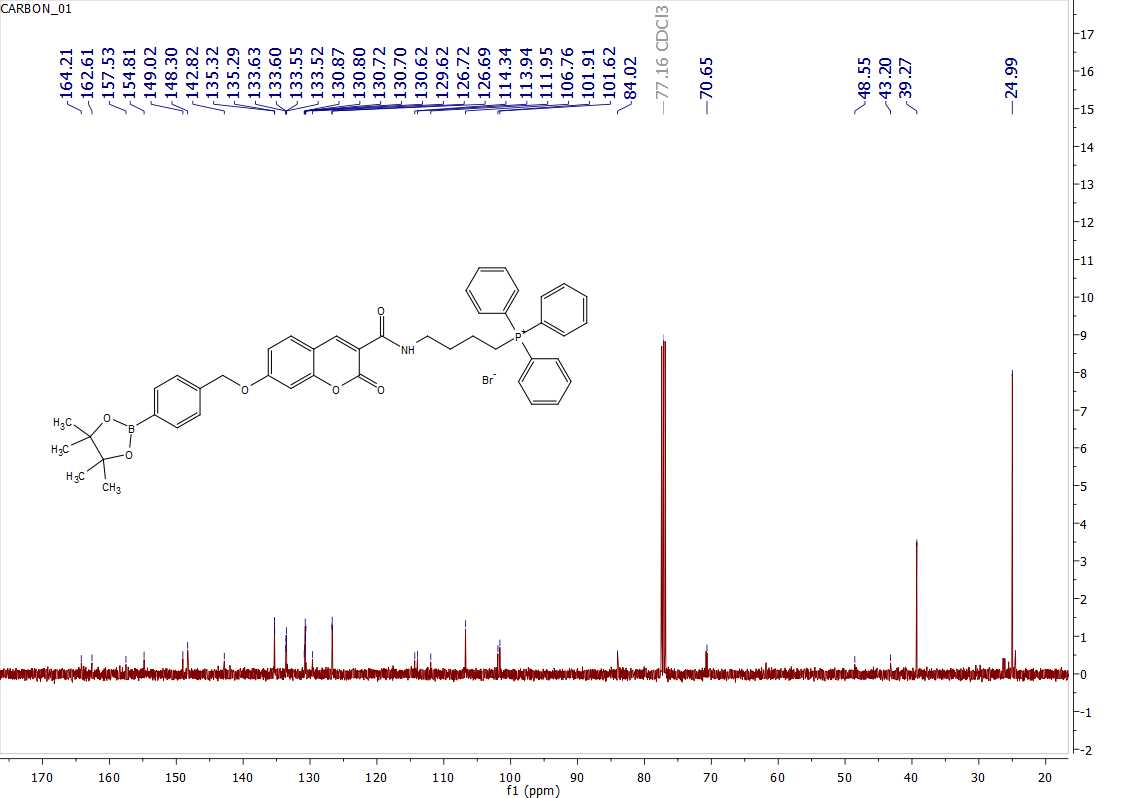 |

Figure S 10: (4-(2-oxo-7-((4-(4,4,5,5-tetramethyl-1,3,2-dioxaborolan-2-yl)benzyl)oxy)-2*H*-chromene-3-carboxamido)butyl)triphenylphosphonium (CM) – ^13^C NMR

| 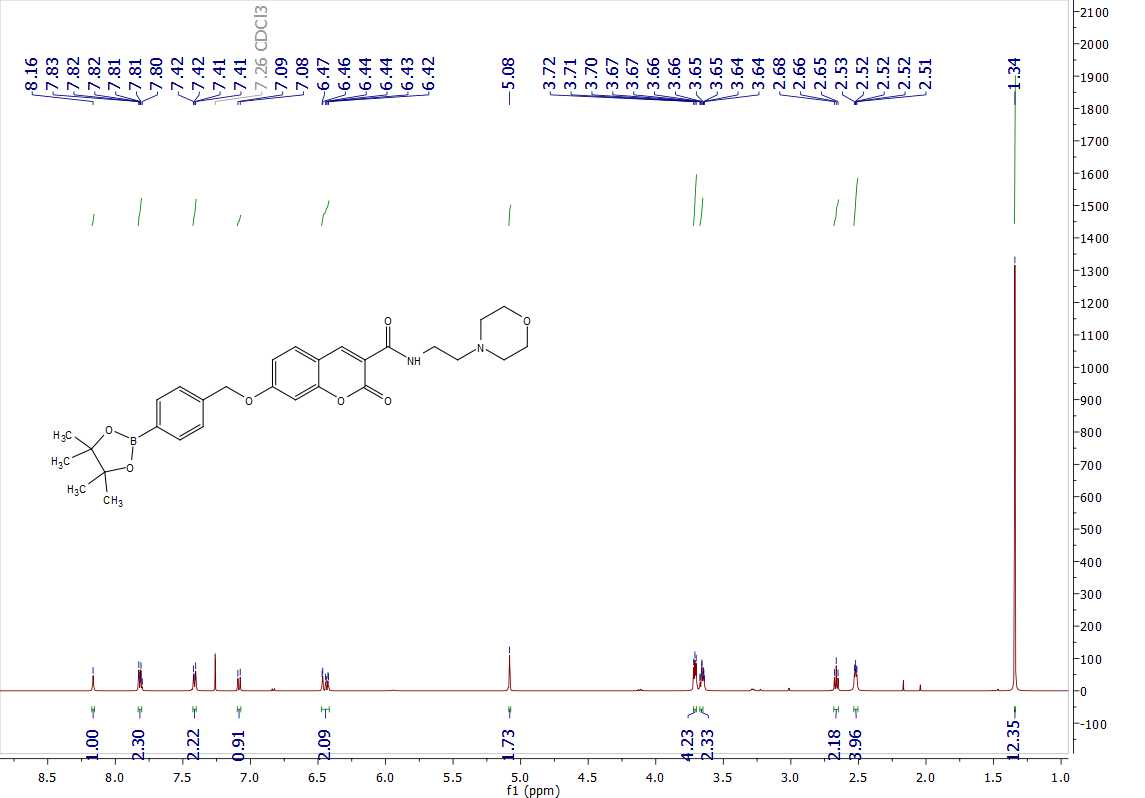 Figure S 11: *N*-(2-morpholinoethyl)-2-oxo-7-((4-(4,4,5,5-tetramethyl-1,3,2-dioxaborolan-2-yl)benzyl)oxy)-2*H*-chromene-3-carboxamide (CL) – ^1^H NMR |
| --- |
| 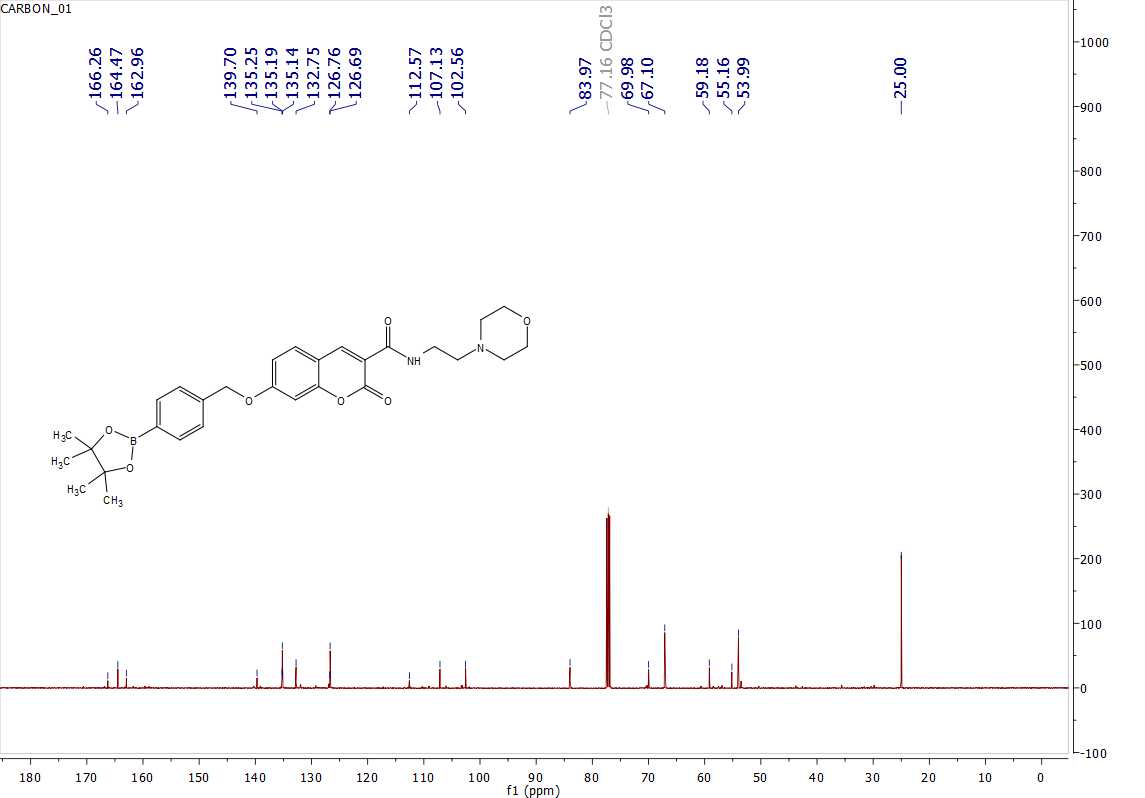 |

**Figure S 12:** *N*-(2-morpholinoethyl)-2-oxo-7-((4-(4,4,5,5-tetramethyl-1,3,2-dioxaborolan-2-yl)benzyl)oxy)-2*H*-chromene-3-carboxamide (CL) – ^13^C NMR

| 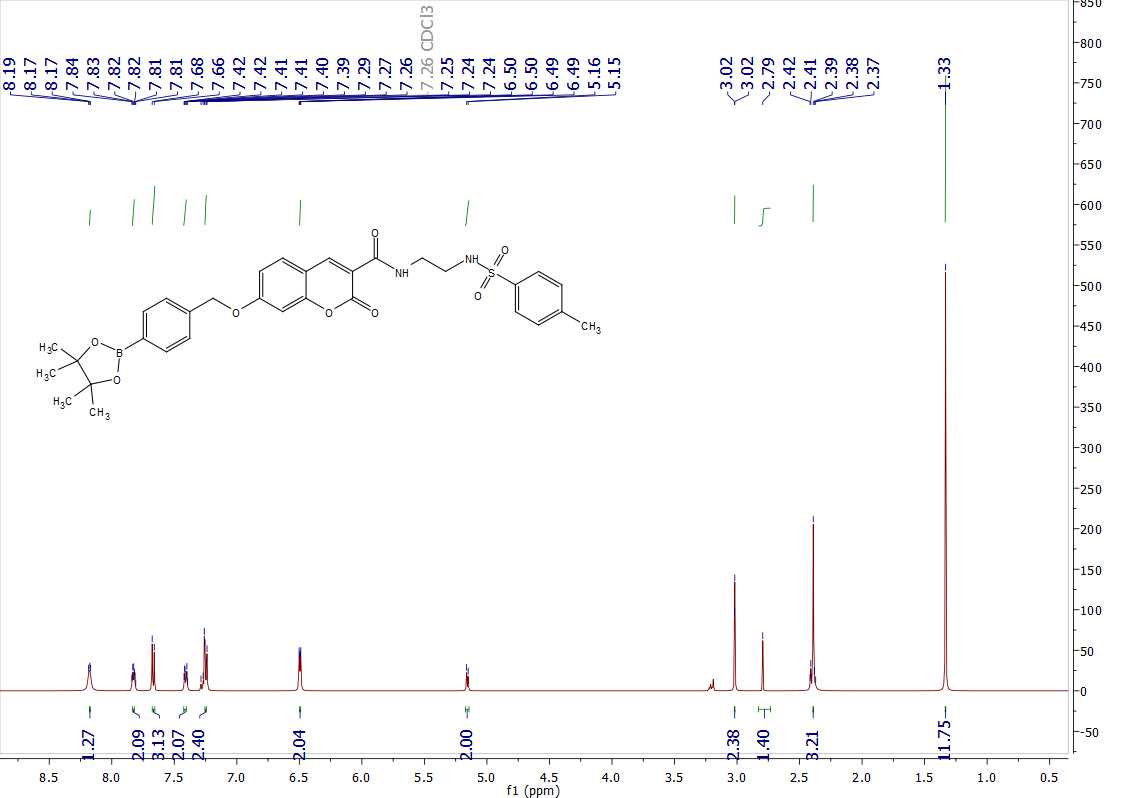 |
| --- |

**Figure S 13:** *N*-(2-((4-methylphenyl)sulfonamido)ethyl)-2-oxo-7-((4-(4,4,5,5-tetramethyl-1,3,2-dioxaborolan-2-yl)benzyl)oxy)-2*H*-chromene-3-carboxamide (CE) – ^1^H NMR

| 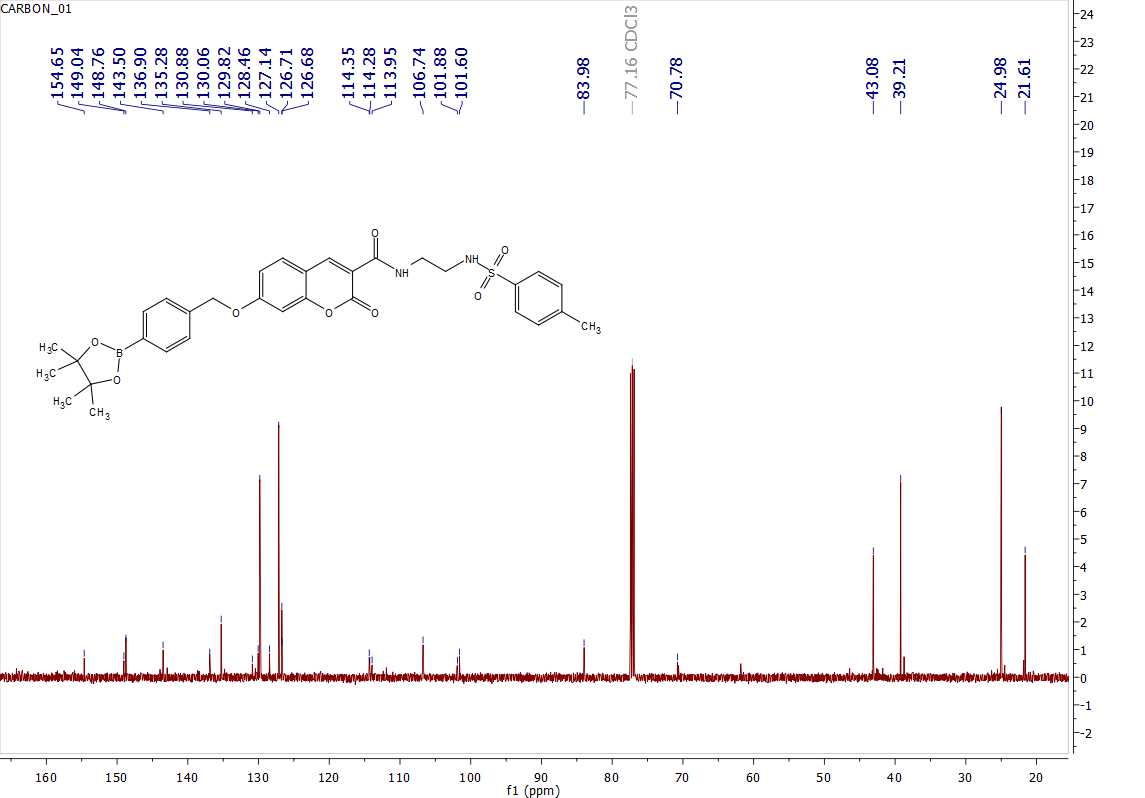 |
| --- |

**Figure S 14:** *N*-(2-((4-methylphenyl)sulfonamido)ethyl)-2-oxo-7-((4-(4,4,5,5-tetramethyl-1,3,2-dioxaborolan-2-yl)benzyl)oxy)-2*H*-chromene-3-carboxamide (CE) – ^13^C NMR\

# Fluorescence

| 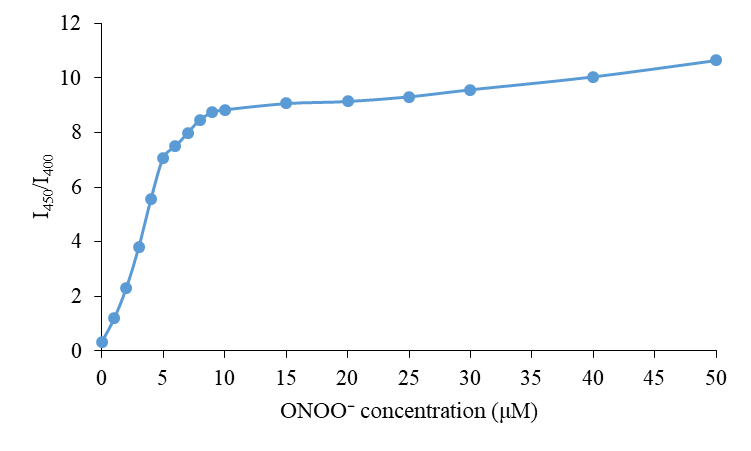 |
| --- |

Figure S 15: Dose dependence curve for CM (5 μM) in the presence of ONOO^-^ (1, 2, 3, 4, 5, 6, 7, 8, 9, 10, 15, 20, 25, 30, 40, 50 μM) in PBS buffer, pH= 7.4 at 25 °C. Fluorescence intensities were measured with λ_ex_ = 340 (bandwith: 20) nm on a BMG Labtech CLARIOstar® plate reader.

| 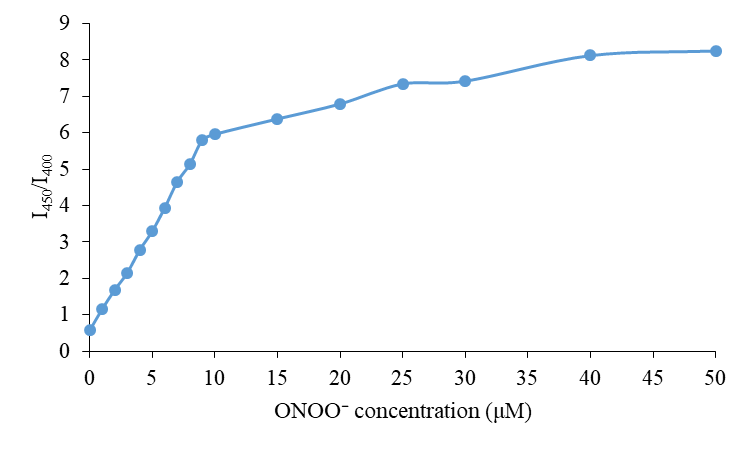 |
| --- |

Figure S 16: Dose dependence curve for CL (5 μM) in the presence of ONOO^-^ (1, 2, 3, 4, 5, 6, 7, 8, 9, 10, 15, 20, 25, 30, 40, 50 μM) in PBS buffer, pH= 7.4 at 25 °C. Fluorescence intensities were measured with λ_ex_ = 340 (bandwith: 20) nm on a BMG Labtech CLARIOstar® plate reader.

| 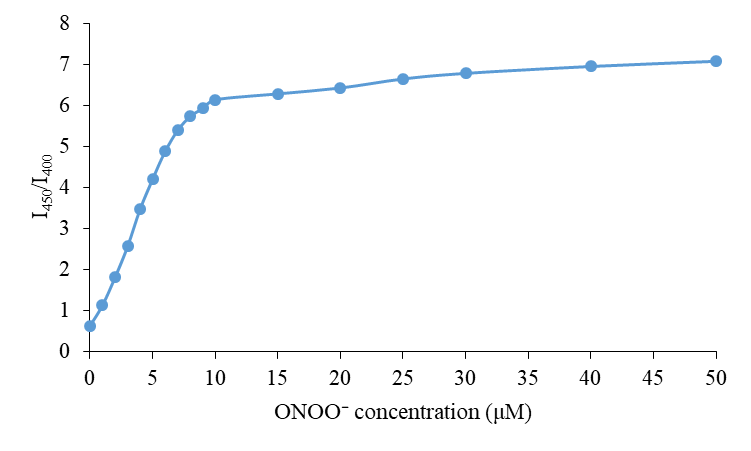 |
| --- |

Figure S 17: Dose dependence curve for CE (5 μM) in the presence of ONOO^-^ (1, 2, 3, 4, 5, 6, 7, 8, 9, 10, 15, 20, 25, 30, 40, 50 μM) in PBS buffer, pH= 7.4 at 25 °C. Fluorescence intensities were measured with λ_ex_ = 340 (bandwith: 20) nm on a BMG Labtech CLARIOstar® plate reader.

| 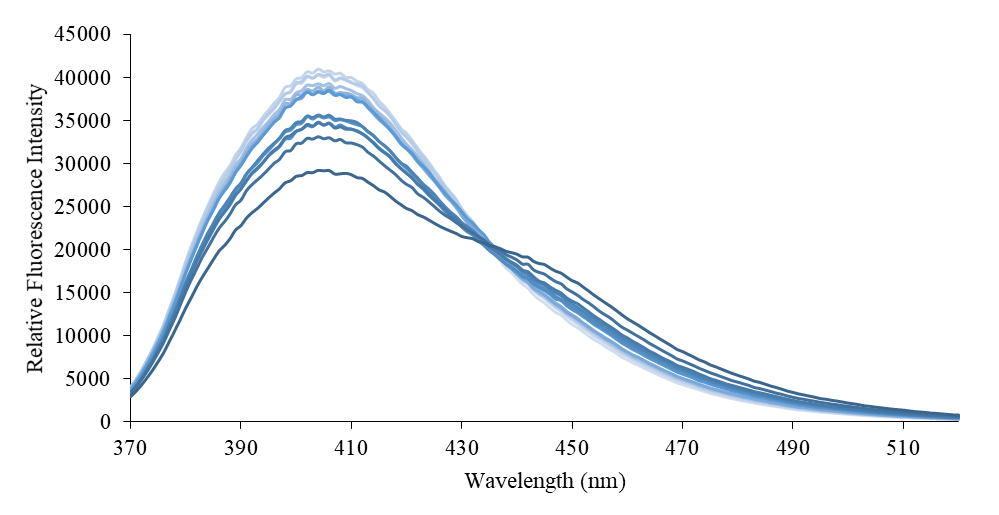 |
| --- |

Figure S 18: Emission spectra for CM (5 μM) in the presence of H_2_O_2_ (0.1, 0.2, 0.3, 0.4, 0.5, 0.6, 0.7, 0.8, 0.9, 1, 1.5, 2 mM) in PBS buffer, pH= 7.4 at 25 °C. Fluorescence intensities were measured with λ_ex_ = 340 (bandwith: 20) nm on a BMG Labtech CLARIOstar® plate reader.

| 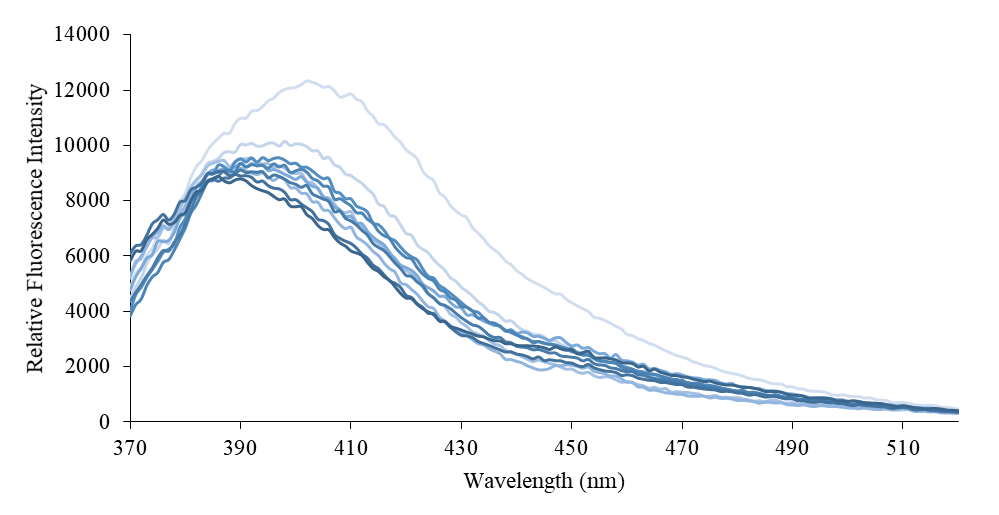 |
| --- |

Figure S 19: Emission spectra for CL (5 μM) in the presence of H_2_O_2_ (0.1, 0.2, 0.3, 0.4, 0.5, 0.6, 0.7, 0.8, 0.9, 1, 1.5, 2 mM) in PBS buffer, pH= 7.4 at 25 °C. Fluorescence intensities were measured with λ_ex_ = 340 (bandwith: 20) nm on a BMG Labtech CLARIOstar® plate reader.

| 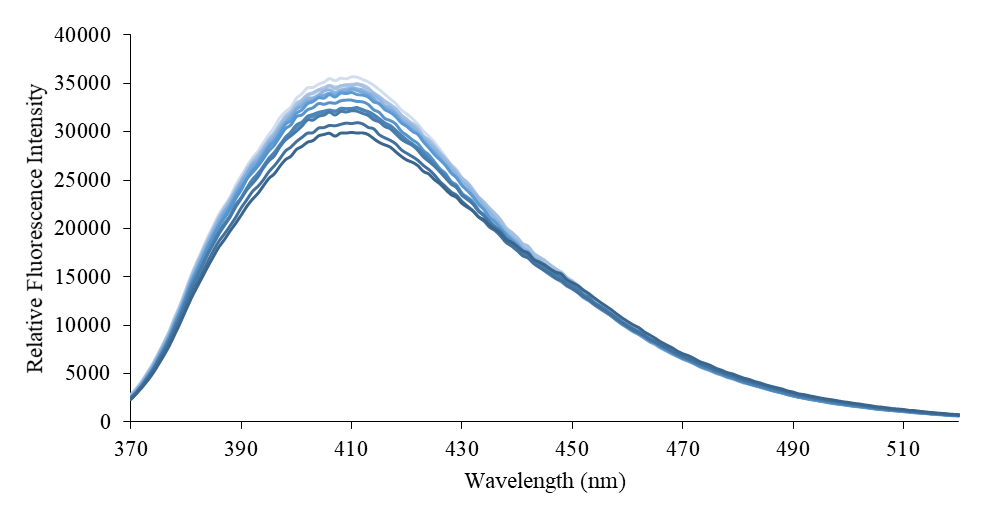 |
| --- |

Figure S 20: Emission spectra for CE (5 μM) in the presence of H_2_O_2_ (0.1, 0.2, 0.3, 0.4, 0.5, 0.6, 0.7, 0.8, 0.9, 1, 1.5, 2 mM) in PBS buffer, pH= 7.4 at 25 °C. Fluorescence intensities were measured with λ_ex_ = 340 (bandwith: 20) nm on a BMG Labtech CLARIOstar® plate reader.

| 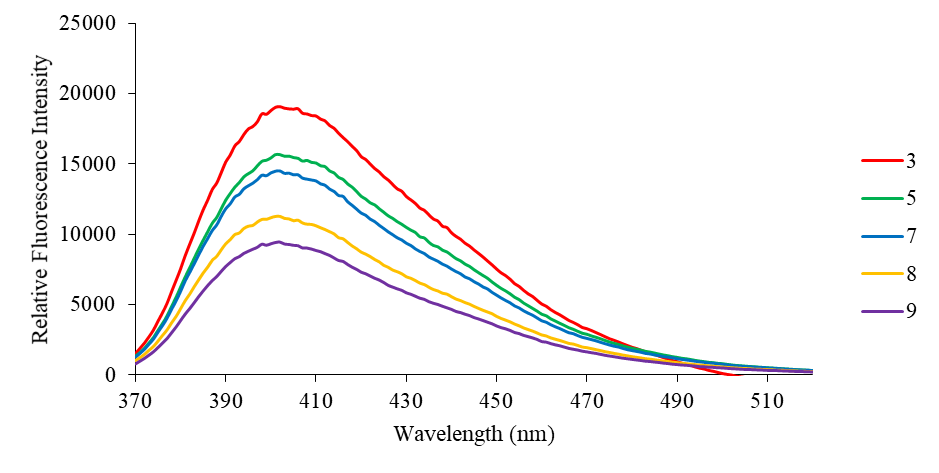 |
| --- |

Figure S 21: Emission spectra for CM (5 μM) at pH 3, 5, 7, 8, and 9 in PBS buffer. Fluorescence intensities were measured with λ_ex_ = 340 (bandwith: 20) nm on a BMG Labtech CLARIOstar® plate reader.

| 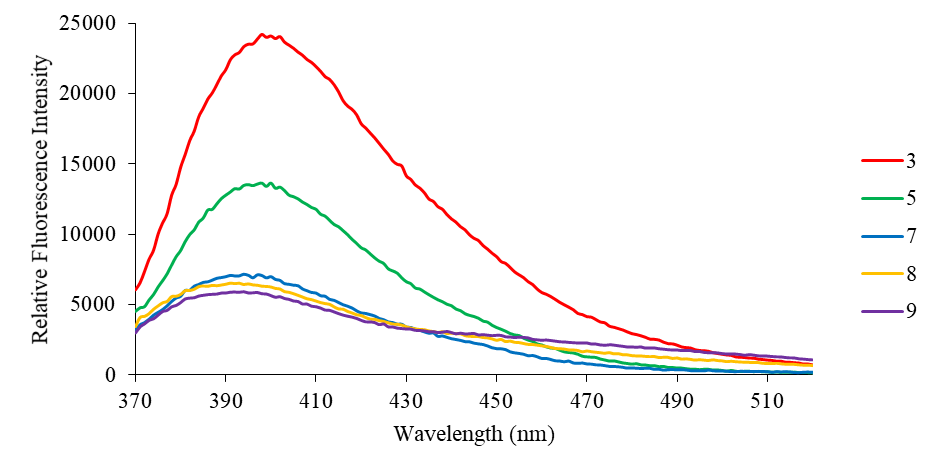 |
| --- |

Figure S 22: Emission spectra for CL (5 μM) at pH 3, 5, 7, 8 and 9 in PBS buffer. Fluorescence intensities were measured with λ_ex_ = 340 (bandwith: 20) nm on a BMG Labtech CLARIOstar® plate reader.

| 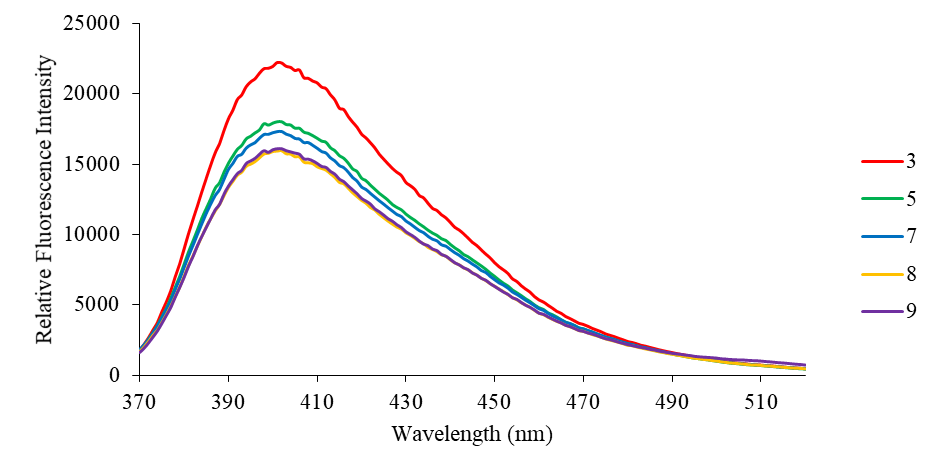 |
| --- |

Figure S 23: Emission spectra for CE (5 μM) at pH 3, 5, 7, 8 and 9 in PBS buffer. Fluorescence intensities were measured with λ_ex_ = 340 (bandwith: 20) nm on a BMG Labtech CLARIOstar® plate reader.

| 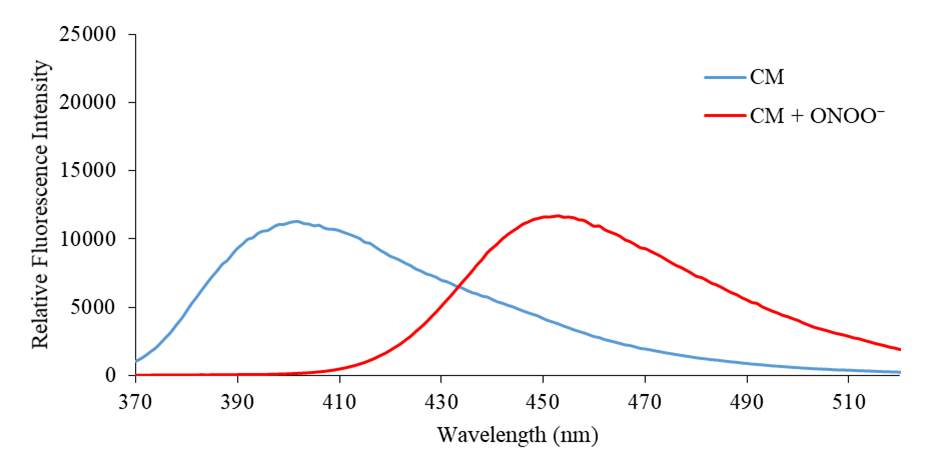 |
| --- |

Figure S 24: Emission spectra for CM (5 μM) without and with ONOO^-^ (50 μM) at pH 8 in PBS buffer. Fluorescence intensities were measured with λ_ex_ = 340 (bandwith: 20) nm on a BMG Labtech CLARIOstar® plate reader.

| 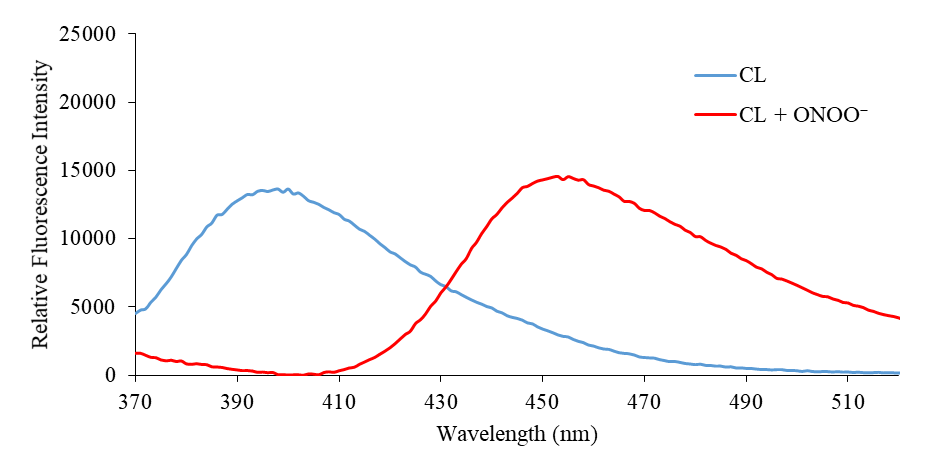 |
| --- |

Figure S 25: Emission spectra for CL (5 μM) without and with ONOO^-^ (50 μM) at pH 5 in PBS buffer. Fluorescence intensities were measured with λ_ex_ = 340 (bandwith: 20) nm on a BMG Labtech CLARIOstar® plate reader.

| 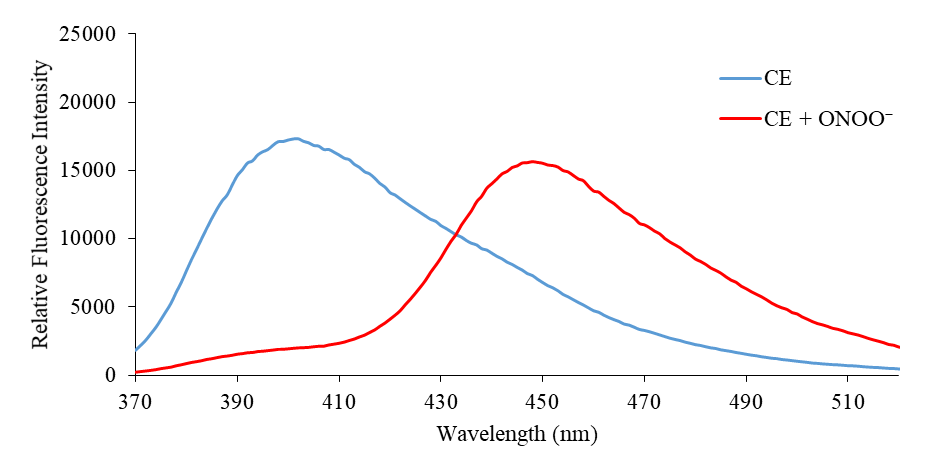 |
| --- |

Figure S 27: Emission spectra for CE (5 μM) without and with ONOO^-^ (50 μM) at pH 7 in PBS buffer. Fluorescence intensities were measured with λ_ex_ = 340 (bandwith: 20) nm on a BMG Labtech CLARIOstar® plate reader.
